# A systematic review and meta-analysis of victimisation and mental health prevalence among LGBTQ+ young people with experiences of self-harm and suicide

**DOI:** 10.1371/journal.pone.0245268

**Published:** 2021-01-22

**Authors:** A. Jess Williams, Christopher Jones, Jon Arcelus, Ellen Townsend, Aikaterini Lazaridou, Maria Michail

**Affiliations:** 1 School of Psychology, Institute for Mental Health, University of Birmingham, Birmingham, United Kingdom; 2 School of Psychology, Self-Harm Research Group, University of Nottingham, Nottingham, United Kingdom; 3 School of Psychology, Centre for Applied Psychology, University of Birmingham, Birmingham, United Kingdom; 4 School of Medicine, Institute of Mental Health, University of Nottingham, Nottingham, United Kingdom; University of Toronto, CANADA

## Abstract

**Background:**

LGBTQ+ youth have higher rates of self-harm and suicide than cisgender, heterosexual peers. Less is known about prevalence of risks within these populations.

**Objectives:**

The first systematic review and meta-analysis to investigate the prevalence of risks among young people throughout the LGBTQ+ umbrella with experiences across the dimension of self-harm, suicidal ideation and suicide behaviour; and how they may differ between LGBTQ+ umbrella groups.

**Data sources:**

MEDLINE, Scopus, EMBASE, PsycINFO, and Web of Science searches were run to identify quantitative research papers (database inception to 31^st^ January, 2020).

**Study eligibility criteria:**

Articles included were empirical quantitative studies, which examined risks associated with self-harm, suicidal ideation or suicidal behaviour in LGBTQ+ young people (12–25 years).

**Synthesis methods:**

2457 articles were identified for screening which was completed by two independent reviewers. 104 studies met inclusion criteria of which 40 had data which could be meta-analysed in a meaningful way. This analysis represents victimisation and mental health difficulties as risks among LGBTQ+ youth with self-harm and suicide experiences. Random-effects modelling was used for the main analyses with planned subgroup analyses.

**Results:**

Victimisation and mental health were key risk factors across the dimension self-harm and suicide identified through all analyses. A pooled prevalence of 0.36 was indicated for victimisation and 0.39 for mental health difficulties within LGBTQ+ young people with experiences of self-harm or suicide. Odds ratios were calculated which demonstrated particularly high levels of victimisation (3.74) and mental health difficulties (2.67) when compared to cisgender, heterosexual counterparts who also had these experiences.

**Conclusions:**

Victimisation and mental health difficulties are highly prevalent among LGBTQ+ youth with experiences of self-harm and suicide. Due to inconsistency of reporting, further risk synthesis is limited. Given the global inclusion of studies, these results can be considered across countries and inform policy and suicide prevention initiatives.

**PROSPERO registration number:**

CRD42019130037.

## Introduction

Worldwide, suicide is one of the leading causes of death for young people [1], with adolescent suicide rates between 11.2–12.7 per 100,000 across low-, middle-, and high-income countries [2]. Suicidal thoughts and attempt are thought to be around 3 times higher among sexual orientation minorities (Lesbian, gay, bisexual, questioning or queer, LGBQ) youth when compared to heterosexual, cisgender counterparts [3]. A recent meta-analysis found suicidal ideation prevalence was demonstrated to be around 28% among gender identity minority groups (transgender and gender non-conforming, TGNC) and suicidal attempt prevalence was 14.8% [4]. Self-harm (defined as self-injury or self-poisoning of self, irrespective of suicidal intent [5]) is known as the most influential risk factor for completed suicide among young people [6,7]. There is also strong evidence that demonstrates the high prevalence of self-harm among young people who identify as LGBTQ+ (Lesbian, Gay, Bisexual, Transgender, Queer or Questioning, and others) [8]. Within LGBQ youth self-harm was reported by 65% of the sample whilst around 46% of TGNC samples have also reported this type of behaviour [9,10].

Among young people generally, regardless of sexual orientation or gender identity, risks associated with experiences of self-harm and suicide are numerous, ranging from childhood neglect to poor academic performance [11,12]. Given this, risk factors are often put into broad categories; demographic, psychosocial, mental health, or psychopathology etc. [13–15]. Within a category such as demographic risks, the individual risk factor can also range widely e.g. age [16], race [17,18] or education level [19]. Additionally, certain populations may also experience risks which are only influential to that specific group of individuals. LGBTQ+ young people are often exposed to additional stressors which are specifically related to their sexual orientation and gender identity when compared to cisgender heterosexual peers, such as institutionalised prejudice, social pressure and victimisation [20–22]. Among the LGBTQ+ umbrella there is also variation of how prevalent a risk may be to a subgroup. For example, someone who is outwardly gender nonconforming may receive more harassments than a cisgender member of the LGBTQ+ umbrella. Therefore, it is possible that there is another layer of risks which TGNC young people face. Gender nonconformity, gender dysphoria, and frustrations due to the long waiting lists for gender affirming medical interventions are common among TGNC populations and have previously been shown to influence suicidal behaviour [23]. Although we know that negative experiences such as institutional prejudice, social pressures, victimisation are associated with self-harm or suicide among those who identify as LGBTQ+ young people [20–22,24], less is known about how prevalent these experiences may be within this population. This systematic review seeks to comprehensively investigate the prevalence of all risks within LGBTQ+ young people who have a history of self-harm, suicidal ideation or attempt. Previous reviews in this population specifically focus on a category of self-harm and suicide; either non-suicidal self-injury or suicide excluding self-harm [25,26]. However, we aim to investigate outcomes across the dimension of self-harm, irrespective of intent, suicidal ideation and attempt to consider differences and similarities within risk prevalence by outcome among LGBTQ+ young people. This will allow us to explore risks across the dimensional structure of self-destructive thoughts and behaviours [27] and consider the comparison of risk across the continuum of suicidal intent. Furthermore, previous reviews have not looked at the prevalence of risk factors for self-harm and suicide across the full LGBTQ+ umbrella, therefore, losing comparability of risks within this broad population [28]. In this study, we consider LGBTQ+ young people as a whole group, and then by sexual orientation minority and gender identity minority groups.

### Objectives

1. To investigate, for the first time, the prevalence of risks associated with the full dimension of self-harm, suicidal ideation or attempts in LGBTQ+ young people who have these experiences.
2. To investigate whether there is a difference in the prevalence of risks between young people who identify as a sexual orientation minority (LGBQ) alongside those who identify as a gender identity minority (TGNC).

## Methods

### Protocol and registration

This review was conducted and reported in accordance with PRISMA guidelines (SM1) [29]. An a-priori protocol was registered on PROSPERO (CRD42019130037), and the full protocol was published in 2019 [30]. As this is a systematic review and meta-analysis of published literature, ethical approval was not sought.

### Search strategy

**Fig 1:**
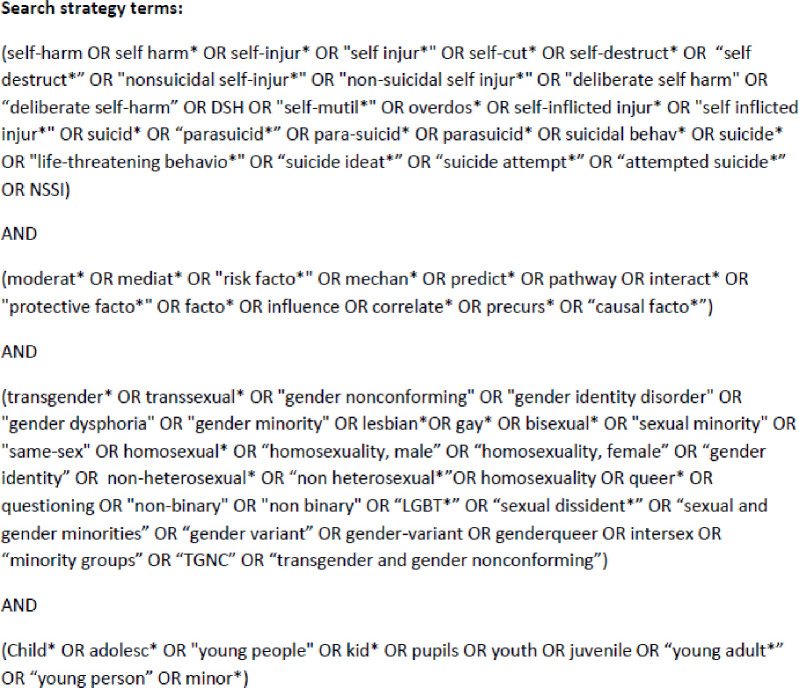
Search strategy terms.

During March 2019, a literature search strategy was developed with an academic skills specialist at the University of Birmingham. An electronic search was conducted on the 31^st^ of March 2019 using MEDLINE, Scopus, EMBASE, PsycINFO, and Web of Science. This was updated on the 31^st^ of January 2020. There was no date limit for identified articles, however only those in English language were considered. Search terms (and their derivatives) focused on the variables of interest; “self-harm”, “suic*”, “adolescent*”, “young person*”, “sexual orientation”, “gender identity” and “risk*”, see Fig 1. The reference list of included articles and key papers within the field were examined for further relevant publications.

### Inclusion criteria

Articles included in this systematic review were empirical quantitative studies, which examined risks across the dimension of self-harm and suicide in LGBTQ+ young people (12–25 years). This age range covers the period of adolescence and early adulthood [31]. An associated risk is operationalised as “an exposure that is statistically related in some way to an outcome” [32; p1], such as significant effect sizes, correlations, mediators, moderators, beta statistics, or any prevalence available relating to an outcome of self-harm or suicide. Mixed-method study designs were included if the quantitative aspects were relevant and extractable. Papers were included if they provided a self-reported or verified group who identified as a sexual orientation or gender identity minority, and any outcome of across the dimension of self-harm and suicide. Studies, whose population were not focused on any sexual orientation or gender identity minorities, were included if they presented information for LGBTQ+ participants separately or if authors were able to offer this information when contacted. Full inclusion criteria are described in Table 1.

**Table 1:** Inclusion criteria used during screening process.

Inclusion Criteria	Exclusion criteria
• Peer reviewed studies. • Any geographical location. • English language. • Empirical quantitative studies, following cross-sectional, prospective, longitudinal, cohort and case-control designs. • Participants that have had a measured outcome from the dimension of self-harm and suicide; self-harm (self-harm or injury to self-irrespective of suicidal intent), suicidal ideation (thoughts, plan, death wish), or suicide attempt (individual took an attempt on their life, suicide death). • Studies must consider risks associated with or predictive of self-harm, suicidal ideation, suicidal attempt or death. • Participants must be young people (12–25 years). • Participants that are identified or self-identified as any sexual or gender minority or member of LGBTQ+.	• Non-peer reviewed literature. • Not English language. • Grey literature such as theses, dissertations or conference proceedings. • Articles such as commentaries, reviews, editorial or opinion pieces. • Empirical qualitative studies. • Participants who have no experience of self-harm, suicidal ideation or suicidal attempt. • Sample not aged between 12 and 25 years, e.g. adults 26 years and above or children 12 years and under. • Participants who are identified as heterosexual or not part of sexual or gender minority.

### Study selection

**Fig 2:**
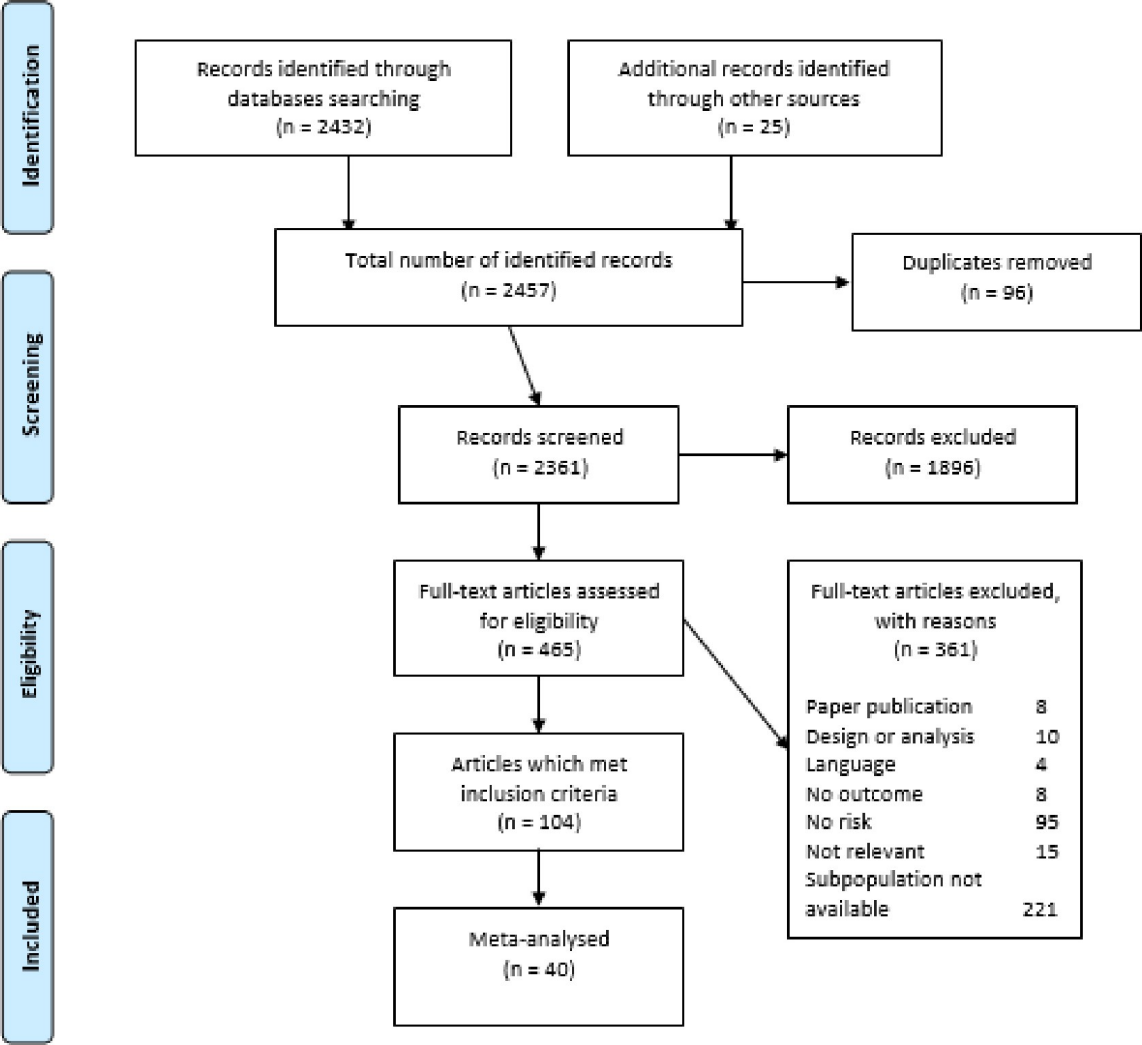
PRISMA flow diagram.

The results of the systematic search are presented in Fig 2. Overall, the searches yielded 2457 results; 96 duplicates were removed. Studies were screened for eligibility at title, abstract and full-text by two independent researchers (AJW and AL) following the PRISMA guidelines [29]. Following the removal of duplications, 2361 were title and abstract screened. If agreement regarding the eligibility of an article could not be met through discussion, a third researcher (MM) was invited to review. This process was repeated at full-text screening for 465 articles, which produced a very high inter-rater reliability (Prevalence- And Bias-Adjusted Kappa, PABAK = 0.948) [33]. This was used due to PABAK being a more stable indicator of inter-rater reliability than Cohen’s Kappa [34].

### Data extraction

A modified version of the data extraction tool used in a previous systematic review was utilised by two independent authors (AJW, AL) to extract data on study design, participants, outcome details, and associated risk [35]. After extraction was completed and checked, any disagreements were discussed and resolved by the research team. Risks were extracted based on a significant relationship to self-harm or suicide outcome. This has the potential to produce multiple reporting of the same study, as the risk may be reporting different outcomes for the same population or the same risk reported for multiple subgroups. For example, within one study, victimisation may be significantly associated with self-harm and suicidal ideation, both of which have an effect size. This would then be extracted twice to yield both sets of information. Initially, outcomes were combined into a single quantitative outcome [36]. Thereby, the overall prevalence of this risk for self-harm and suicide could be observed. Further analysis considered the risk to each outcome individually. The inclusion of multiple reporting from a single study may have resulted in a reduction in confidence intervals for the random effects model as the sample sizes will be included numerous times.

### Risk of bias assessment

To assess quality within the literature, variations of the Newcastle-Ottawa Scale (NOS) were employed [35–38]. This allowed a number of study designs to be considered and assessed. The forms assess risk of bias based on three core aspects of study design: participant selection, comparability of participants, and exposure ascertainment. These were adapted for this systematic review (see SM3), and rated as either low, moderate or high quality using the same category distinctions as previous research [39]. The two reviewers assessed the quality of studies independently, with intermediate agreement (PABAK = 0.43). Agreement was achieved through discussion.

### Data synthesis

The search strategy yielded 104 primary articles, across 102 studies. Given the large number of individual risk factors, similar variables were categorised resembling the format used by previous literature [40]; demographic, psychosocial, mental health difficulties. Rather than use “psychiatric or mental health” however, mental health difficulties was selected due to self-report measures commonly being used, the inclusion of symptomology, and limited information regarding diagnosis of mental health conditions. Additionally, two categories of risk were created, victimisation and LGBTQ+ specific risks. Victimisation includes individual measures which considered the process of the LGBTQ+ young person being treated poorly, harassed, abuse or discriminated against or subjected to bullying. LGBTQ+ specific risks included risks which were strongly related to the LGBTQ+ identity held by the young person, e.g. coming out stress [41], parent being unaware of sexual orientation [42], or negative attitudes towards homosexuality [43]. Risks were classed as victimisation if they suggested direct negative action against the individual, e.g. discrimination, bullying, harassment or threat. Victimisation was selected as representative title as it most often occurred within the studies. Risks which were both victimisation and LGBTQ+ specific, such as trans, bi, and homophobic bullying, were categorised as victimisation.

There was a large amount of inconsistency among individual risks for three categories: demographic, psychosocial and LGBTQ+ specific risks. This did not allow for meaningful clustering of variables into meta-analysis which would provide a prevalence of risk among LGBTQ+ young people who had experiences of self-harm or suicide. Furthermore, numerical evidence was not available for many individual risks; in these instances, either there was no statistically significant statistics available for associated risks, effect sizes, correlations, mediators, moderators, beta statistics, or any reporting of prevalence. Numerical data was predominantly available within victimisation and mental health difficulties; therefore these risks were analysed. The 65 studies not included in meta-analysis due to are briefly described by risk category, and separated by population (e.g. sexual orientation minority, gender identity minority, LGBTQ+ umbrella).

### Numerical analysis

A meta-analysis was conducted for two risks associated with self-harm and suicide among LGBTQ+ young people; victimisation and mental health difficulties, where sufficient data for aggregation were available. For these two risks, outcome data from forty primary studies were synthesised. The purpose of the meta-analysis was to 1) to investigate the prevalence of victimisation and mental health difficulties associated with self-harm, suicidal ideation or suicidal attempt among LGBTQ+ young people with these experiences; 2) to investigate whether there is a difference in the prevalence of victimisation and mental health difficulties among those young people who identify as a sexual orientation minority (LGBQ) and those who identify as a gender identity minority (TGNC); 3) to identify whether the prevalence of victimisation and mental health difficulties is different in LGBTQ+ young people who have experiences of self-harm, suicidal ideation or attempt compared with cisgender heterosexual young people with these experiences.

Event rates of primary studies were log transformed before numerical syntheses such that they were all the same unit of measure (but back-transformed for clear presentation in tables). Studies with an event rate of zero or one were excluded from analysis as studies with a small sample size do not permit accurate estimations of event rate. Where data was available for the target population subgroup and a control subgroup of cisgender and heterosexual individuals, odds ratios were calculated.

The random effects model was used as this assumes that not all studies have the same power to detect effects, therefore, a common effect size cannot be assumed. As the study effects were normally distributed, the DerSimonian and Laird method was selected to determine the variation between the studies to fit the random effects model [44]. The random effects model was extended to include explicit consideration of the methodological quality of the primary studies. This “quality effects model” (QEM) used the NOS total score to characterise the overall quality of the study. This QEM model can be interpreted as the meta-analytic synthesis that would have been obtained if all the studies had been of the same methodological quality as the highest rated study within the review, thereby providing a measure of attenuation to the methodological variation of included studies.

Higgins I^2^ was used to determine the level of heterogeneity within the primary studies with a value of above 75% considered problematic. Sensitivity analysis was conducted to identify studies disproportionately influencing results. Such studies were excluded from subsequent analyses due to the high risk of bias. Subgroup analysis was also used to aid the identification of sources of problematic heterogeneity.

Publication bias and small study effects were also estimated by inspection of funnel plots. In absence of publication bias, high precision studies will be evidenced near the average, with lower precision studies spread evenly and symmetrically on both sides of the average, creating a funnel-shaped distribution. Publication bias is indicated by the absence of studies in the area of the final plot associated with small (i.e. non-significant) effect sizes in small studies.

If publication bias was evidenced then a trim and fill procedure was undertaken. This produced an adjusted effect size (controlling for publication bias), and the impact of publication bias was assessed by comparison with the uncorrected random effects model. The fail-safe N was also calculated using the Orwin algorithm [45]. This is the estimation of missing studies that was required to render the effect non-significant. If the fail-safe N is large (in relation to the number of studies included in the synthesis), then the synthesis could be considered robust to the effects of publication bias.

Before searches were conducted, two a-priori hypotheses were established to consider heterogeneity which may occur within the data [30]. The first suggested that heterogeneity may be explained by consideration of sexual orientation (LGBQ) and gender identity minorities (TGNC) as separate populations. This allows us to determine whether there are similar levels of risk within both groups. The second a-priori aim was to consider risk by age group; however, this was not possible given the final dataset. Additionally, a subgroup analysis was run based on the type of outcomes reported: self-harm, suicidal ideation, and suicidal attempt. Summary effects and associated heterogeneity measures were calculated for each subgroup, the significance of difference between these being evaluated by the comparison of their 95% confidence intervals.

## Results

One-hundred and four papers from 102 studies were included, which met all the inclusion criteria and contained extractable significant risks associated with self-harm, suicidal ideation, or suicidal attempt. Twenty-six studies examined a form of self-harm (e.g. self-harm with suicidal intent, self-harm intent unspecified, non-suicidal-self-injury) whereas 77 considered ideation and 76 considered behaviour, studies often considered more than one outcome. None of the studies included information on participants who died by suicide. Two of the included papers [46,47] utilised the same dataset as a previously included study [48,49]. These were included as separate papers, given that they highlight risk factors which the primary study did not. The majority of studies were cross-sectional (n = 91); with 10 longitudinal studies, and 3 cohort studies. A total of 1,146,395 participants were included, with 129,469 (11.3%) being LGBQ and 13,041 (1.1%) being TGNC. Ages ranged from 12–25 (M = 17.7, SD = 1.9). Studies were mainly based within the U.S.A (n = 77), followed by the U.K. (n = 7), and China (n = 4). For full individual study characteristics, see supplementary materials 4 tables A [17,18,41,43,49–109] and B [16,23,42,47,110–144] (SM3) in S1 Table. From this document, further figures regarding heterogeneity and influential studies are also available.

From the 104 included papers, 64 were unable to be numerically synthesised [17,18,41,43,49–109]. The individual characteristics of these studies can be seen in Supplementary Table A (SM3) in S1 Table. The population of these papers represented a total of 929,802 individuals, of whom 90,767 were LGBTQ+ identifying (9.76%). Therefore, these studies are considered 81.1% of the overall population. These studies did evidence multiple risks associated with experiences of self-harm and suicide among LGBTQ+ young people. The individual risk factors were varied and numerous to the extent that they could not be individually considered in relation to prevalence. However, by categorising these broadly, some information can be gained.

Most of the papers which were not numerically synthesised, focused on samples which only considered sexual orientation minorities, see Table 2. With fewer studies examining TGNC populations or across the LGBTQ+ umbrella. Across all populations, psychosocial risks were most commonly cited in associated with self-harm and suicide. Victimisation and mental health difficulties were evident, although without reinforcing numerical evidence.

**Table 2:** Risks associated with experiences of self-harm or suicide among LGBTQ+ young people: Data unable to be numerically synthesised.

**Categories of risk**	**LGBQ k = 48 N (%)**	**TGNC k = 8 N (%)**	**LBGTQ+ k = 8 N (%)**
**Demographic variables** (*e*.*g*. *natal gender*, *age*, *race*)	15 (30.6)	4 (50)	3 (37.5)
**Psychosocial variables** (*e*.*g*. *low self-esteem*, *dating violence*, *suicide of friend or family*, *abuse*)	31 (63.3)	4 (50)	5 (62.5)
**Victimisation variables** (*e*.*g*. *LGBTQ hate crime*, *homophobic bullying*, *school bullying*, *cyber bullying*)	27 (55.1)	2 (25)	4 (50)
**Mental health difficulties variables** (*e*.*g*. *depression*, *substance use*, *bipolar*, *anxiety*)	10 (20.4)	4 (50)	2 (25)
**LGBTQ+ specific variables** (*e*.*g*. *gender-role nonconformity*, *internalised homophobia*, *parental rejection*, *loss of friends due to sexual orientation*)	13 (26.5)	2 (25)	3 (37.5)

### 1. Meta-analysis: Victimisation

A random effects model was calculated, using the generic inverse variance method, to examine the prevalence of victimisation as a risk associated with experiences of self-harm, suicidal ideation or suicidal attempt among LGBTQ+ young people. Sixty-three estimates from 31 individual samples were reported, representing 331,321 participants in total. The random effects models reported a pooled prevalence estimate of 0.33 and a 95% confidence interval of between 0.29–0.38 among LGBTQ+ young people with self-harm or suicide experiences.

A high level of between study variation (heterogeneity) could not be attributed to differences in individual reaction to victimisation within the included studies (Higgin’s I^2^ = 99%). Therefore, the prevalence estimates of the primary studies may be influenced by the presence of uncontrolled or confounding factors. Given this substantial level of heterogeneity, the impact of disproportionately influential individual studies was assessed using a leave-one-out analysis. Following this, Taliaferro and Muehlenkamp (2017) [137] was removed from the meta-analysis [137]. This was due to a variable being extracted multiple times as numerical data was given per sexual orientation, this resulted in a large volume of included variables. Therefore, this study was overtly overrepresented within the sample.

**Fig 3:**
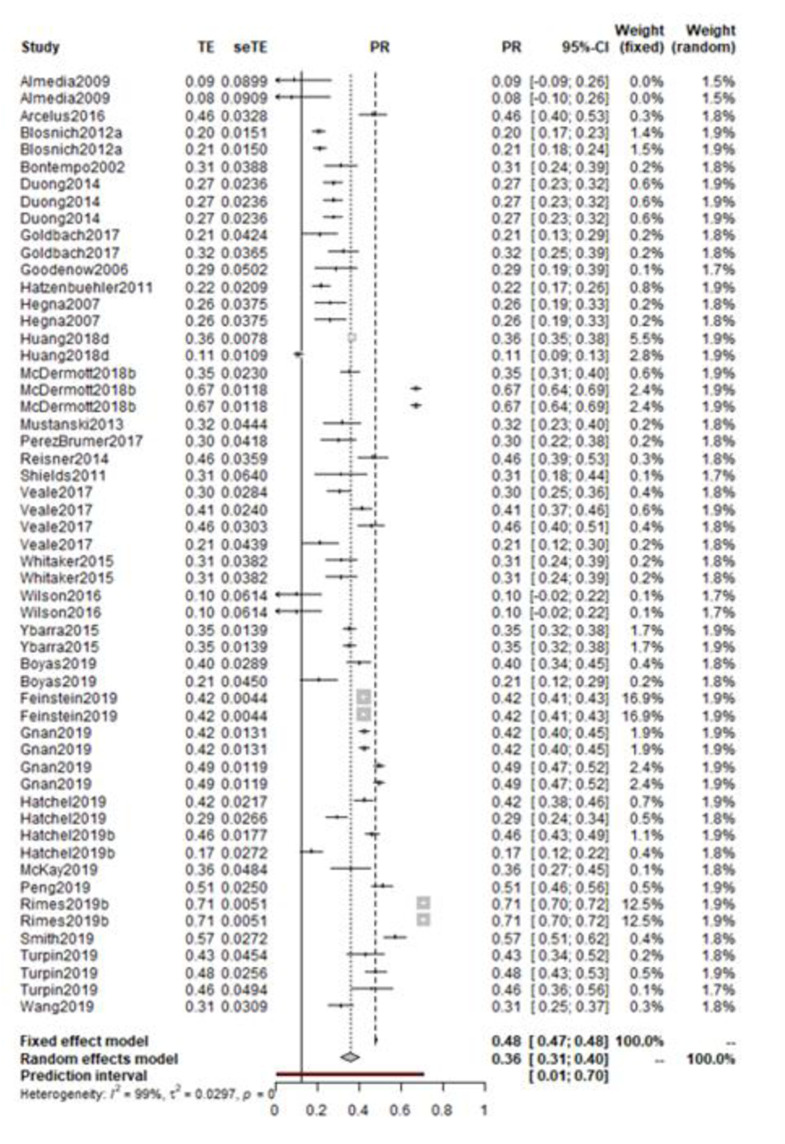
Forest plot of victimisation prevalence among LGBTQ+ with experiences of self-harm or suicide.

The random effects model was recalculated with 55 measures of prevalence from 30 unique samples. The corrected random effects model reported a pooled prevalence estimate of 0.36 (95%CI: 0.31–0.40) (Fig 3). The corrected random effects model did not impact heterogeneity (Higgin’s I^2^ = 99%). Accordingly, the observed heterogeneity could not be considered to be the result of overly influential individual studies, and therefore other sources of heterogeneity require exploration.

The Quality Effects Model was calculated using the total score from the risk of bias ratings, (individual study ratings can be found in SM3). The QEM can be interpreted as the meta-analytic synthesis that would have been obtained had all the studies been of the same methodological quality as the best study within the review. This reported an estimate of 0.36 (95% CI: 0.31–0.41). Given the similarity between the random effects model and the synthesis derived from the quality effects model, it is possible to conclude that the ratings of methodological quality did not have a significant and substantial impact upon the estimates of prevalence.

Visual inspection of the funnel plot of victimisation prevalence there is little evidence of publication bias. A fail-safe number of 107 suggested that an additional 101.9% of the existent literature would be required for unpublished null effects for the meta-analytic effect to become non-significant. Thus, the observed effect is considered robust to publication bias.

To further assess the impact of methodological variation upon heterogeneity, a series of subgroup analyses were conducted (Table 3). The first considered risk of bias ratings; low, moderate, and high quality (Q = 19.5, p < 0.01). Both high-rated and low-rated studies evidenced higher prevalence than those rated as moderate quality.

**Table 3:** Subgroup analyses of victimisation prevalence among LGBTQ+ young people with self-harm or suicidal experiences.

	Number of estimates (N)	Prevalence Rate	95% CI	Q	I^2^ (%)	Σ^2^	Q, df, p
**QUALITY RATING**							Q = 19.50, df = 2, p = 0.01
**Low**	7	0.46	0.34–0.58	347.88	98.3	0.02	
**Moderate**	31	0.28	0.24–0.32	686.32	95.6	0.01	
**High**	17	0.45	0.37–0.52	4107.33	99.6	0.02	
**POPULATION**							Q = 0.11, df = 1, p = 0.74
**LGBQ**	27	0.34	0.27–0.42	6282.68	99.6	0.03	
**TGNC**	9	0.33	0.24–0.41	108.99	92.7	0.01	
**OUTCOME**							Q = 12.18, df = 2, p = 0.01
**Self-harm**	10	0.39	0.31–0.48	353.09	97.5	0.02	
**Suicidal ideation**	21	0.35	0.33–0.38	212.38	93.4	0.00	
Suicidal attempt	15	0.26	0.20–0.31	212.38	93.4	0.01	

Subgroup analysis was utilised to explore the impact of uncontrolled covariates upon victimisation. Initially, this evaluated differences in prevalence of victimisation between groups of sexual orientation (LGBQ) or gender identity groups (TGNC) with these experiences of self-harm and suicide. This analysis was to explore whether a particular identity group experiences greater victimisation than others. Studies which combined the populations or looked at just one representation of LGBQ were excluded from this analysis. The subgroup analysis showed that prevalence rates of victimisation were relatively consistent across all gender identity and sexual orientation studies/groups (Q = 0.11, p = 0.74). However, heterogeneity was notably lower within the TGNC studies. This may be related to a small number of studies being included, as analysis of LGBQ triples the study sample. Following this, subgroup analysis was conducted regarding outcome. Again, studies were excluded if they collapsed two distinct categories; suicidal ideation and suicidal attempt. Studies with self-harm as outcome demonstrated an overall victimisation prevalence rate of 39%. This suggests that higher rates of victimisation are associated with self-harm when compared to suicidal thoughts or attempts among LGBTQ+ participants.

The prevalence of victimisation within LGBTQ+ young people with experiences of self-harm or suicide was compared to matched cisgender, heterosexual control counterparts. These young people also had experiences of self-harm or suicide. The odds ratios (19 estimates from 12 studies) were synthesised using the generic inverse variance. An odds ratio of 4.82 (CI: 3.67–6.32) was reported. Between studies heterogeneity was high (I^2^ = 98%) suggesting uncontrolled methodological or conceptual factors contributing variations in reported risks. Therefore, a leave-one-out analysis was conducted to identify studies that might be exerting a disproportionate influence on the overall meta-analysis. One study was identified as both heterogeneous and influential, demonstrated by a change of effect of over 13%. Thus, Turpin and colleagues’ study was removed to give a more conservative overall odds ratio [139].

**Fig 4:**
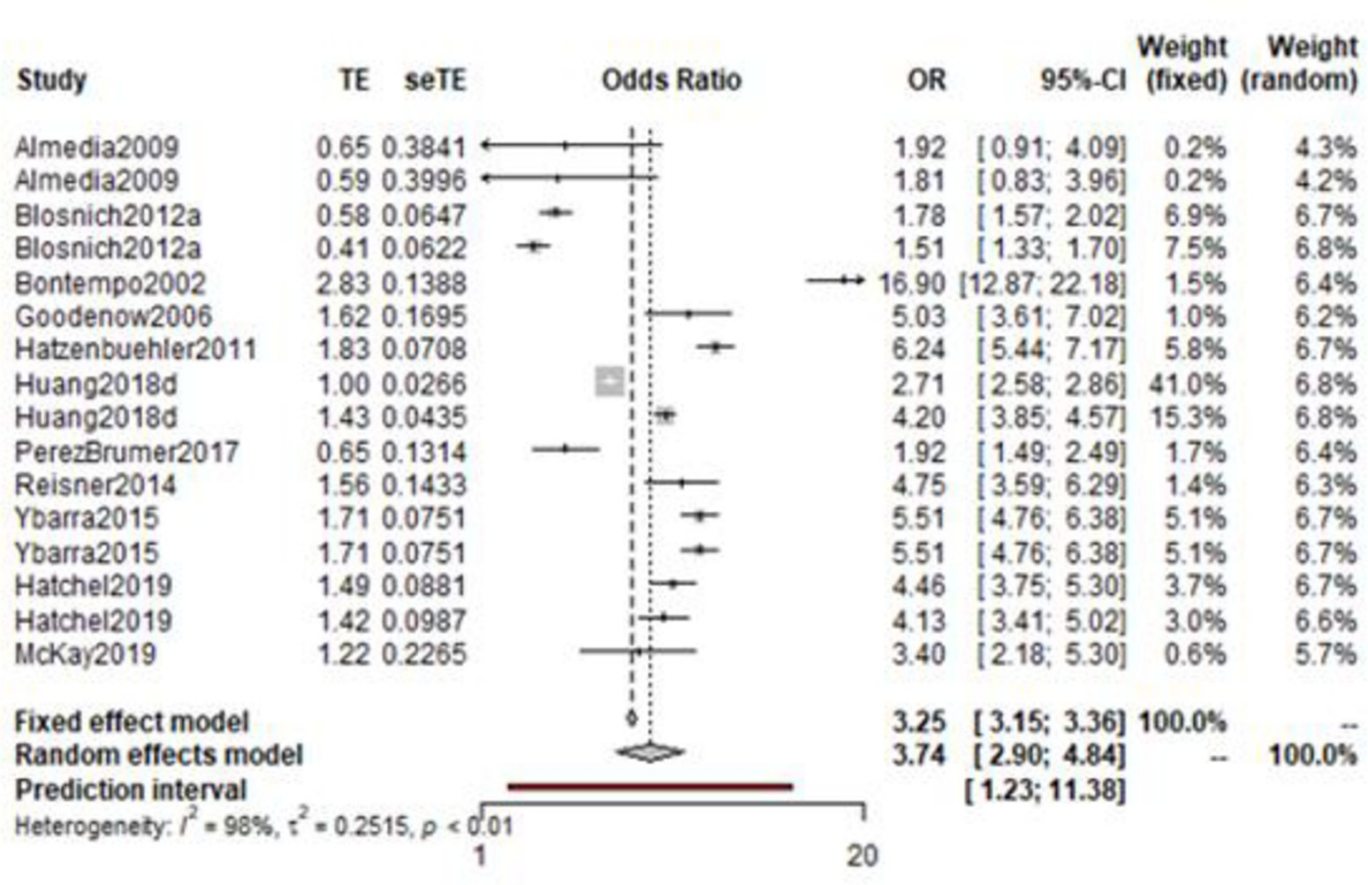
Odds ratio among LGBTQ+ young people with experiences of self-harm or suicide compared to cisgender, heterosexual young people with experiences of self-harm or suicide.

The following meta-analysis was based on the remaining 16 odds ratios from 12 studies. This produced a synthesised odds ratio of 3.74 (95% CI: 2.90–4.84)(Fig 4). The corrected random effects model produced very little change to the heterogeneity level, (Higgin’s I^2^ = 98%). Given the small number of studies, further analyses including an assessment for publication bias were not feasible.

### 2. Meta-analysis: Mental health difficulties

**Fig 5:**
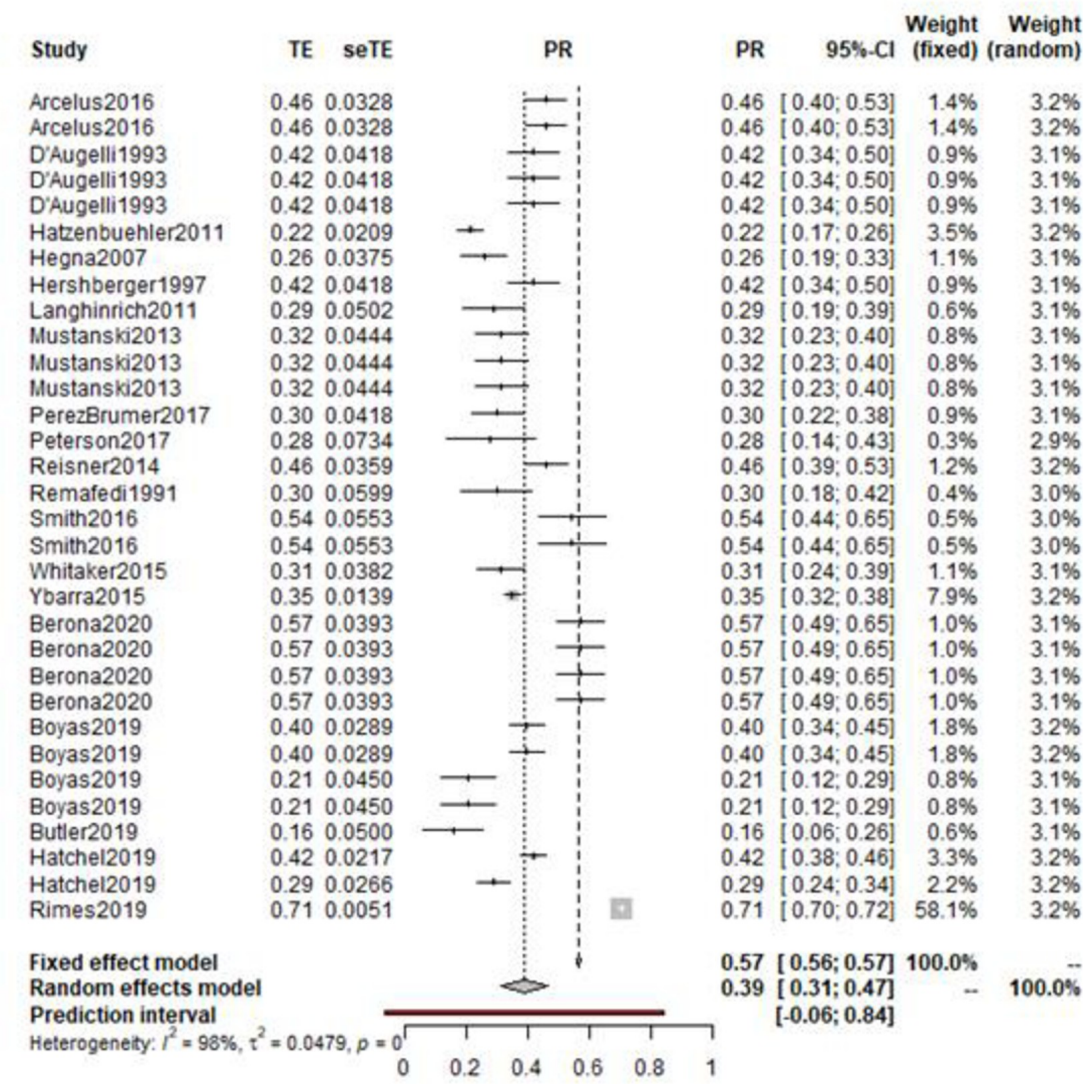
Overall prevalence of mental health difficulties within LGBTQ+ young people with experiences of self-harm or suicide.

A second random effects model was calculated to consider the prevalence of previous mental health difficulties within LGBTQ+ young people who have an experience of self-harm, suicidal ideation or suicidal attempt. A total of 166,810 participants were assessed over 22 studies which produced 51 estimates. The model calculated a prevalence of mental health difficulties of 0.36 (95% CI: 0.29–0.43). Again, a high level of heterogeneity was found (Higgin’s I^2^ = 99%). A leave-one-out analysis was therefore run, with the influential studies being evaluated for inclusion. Studies were omitted if they disproportionally influenced the overall result [136–138]. The random effects models were then recalculated with the 19 studies and 32 variables. This resulted in the prevalence of mental health difficulties increasing to 0.39 (95% CI: 0.31–0.47) (Fig 5). While high heterogeneity remained (Higgin’s I^2^ = 98%).

Visual observation of a funnel plot and trim-and-fill procedure suggests the absence of publication bias. Following Orwin’s algorithm, it was shown that 31 unpublished null studies would be needed to reduce the meta-analytic effect found within this sample. Again, subgroup analyses considering the risk of bias were conducted. The QEM model reported an estimate of 0.39 (95% CI: 0.31–0.47), suggesting that there were not enough differences regarding the risk of bias ratings to substantially influence the overall effects. Subgroup analysis of this sample demonstrated that 4 studies were considered high quality, 14 were of moderate quality and 3 of low quality. However, little could be concluded from between groups differences (Q = 1.54, P = 0.46).

Further subgroup analyses were conducted to investigate the impact of uncontrolled covariates relating to mental health difficulties prevalence (Table 4). The first of these again considered the prevalence differences which may occur between LGBQ and TNGC samples. This analysis evidenced that LGBQ young people were shown to have a higher prevalence of mental health difficulties than TGNC individuals (42% vs 34%). The difference in effect size is likely related to the large difference of included studies. The Higgins I^2^ value for both groups were still high, suggesting that these studies do contribute to heterogeneity, although to lesser extent within TGNC populations. A similar subgroup analysis regarding outcome was conducted, this demonstrated that the rates of mental health difficulties were slightly more prevalent among those with suicidal ideation.

**Table 4:** Subgroup analyses of mental health difficulties prevalence among LGBTQ+ populations who have experiences of self-harm or suicide.

	Number of estimates (N)	Prevalence Rate	95% CI	Q	I^2^ (%)	Σ^2^	Q, df, p
**QUALITY RATING**							Q = 1.54, df = 2, p = 0.46
**Low**	11	0.41	0.33–0.49	122.06	91.8	0.01	
**Moderate**	17	0.36	0.31–0.41	125.83	87.3	0.00	
**High**	4	0.47	0.25–0.69	417.38	99.3	0.05	
**POPULATION**							Q = 2.43, df = 1, p = 0.30
**LGBQ**	20	0.42	0.32–0.53	1227.71	98.5	0.05	
**TGNC**	5	0.34	0.22–0.45	37.56	89.4	0.01	
**OUTCOME**							Q = 0.41, df = 2, p = 0.82
**Self-harm**	3	0.38	0.20–0.53	30.19	93.4	0.02	
**Suicidal ideation**	8	0.40	0.35–0.44	32.70	78.6	0.00	
Suicidal attempt	19	0.38	0.31–0.44	222.21	91.9	0.02	

**Fig 6:**
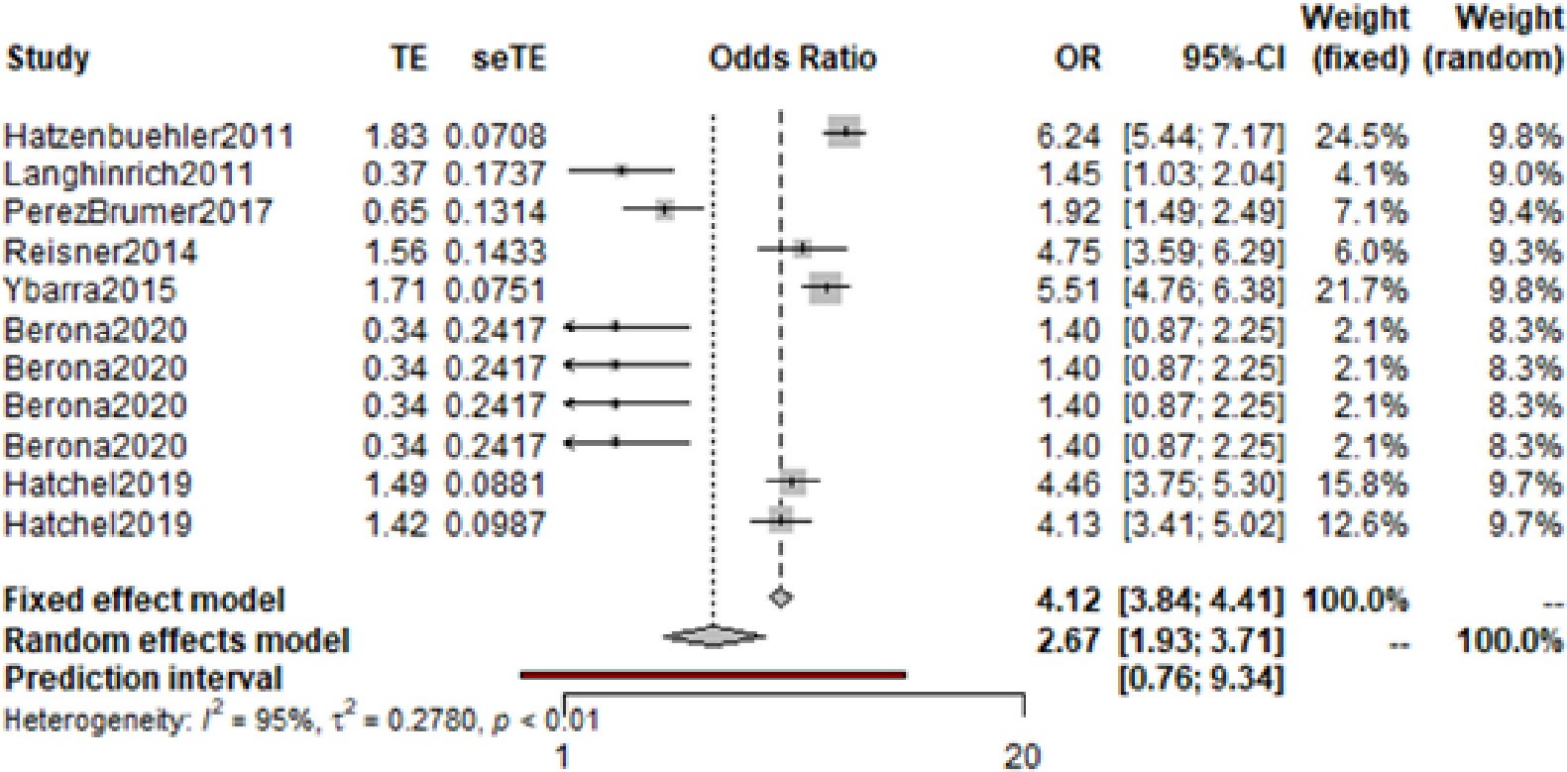
Odds ratio of LGBTQ+ young people with experiences of self-harm or suicide compared to cisgender, heterosexual peers with experiences of self-harm or suicide.

Following this, a meta-analysis of odds ratios was conducted; considering prevalence of mental health difficulties among LGBTQ+ young people and cisgender, heterosexual young people both with experiences of self-harm or suicide. Only 7 studies had available data. The random effects model calculated an odds ratio of 2.67 (95% CI: 1.93–3.71), with a high level of heterogeneity (I^2^ = 95%) (Fig 6). However, due to the limited number of studies, further analysis was not conducted.

## Discussion

This is the first meta-analysis which evidences prevalence of victimisation and mental health difficulties within young people aged 12–25 who identify as LGBTQ+ with experiences of self-harm, suicidal ideation and attempt. The review consisted of 142,510 participants who were a sexual orientation or gender identity minority. Due to limited information reported within the studies, it was not possible to consistently consider TGNC participants by their sexual orientation as well. Evidence demonstrated high prevalence of victimisation (36%) and mental health difficulties (39%) within these populations. Our review shows that these experiences were respectively 3.74 times and 2.67 times higher among young LGBTQ+ people than their cisgender, heterosexual counterparts. There were only 10 studies which were considered high-quality, with most studies (81%) being rated as moderate quality. Substantial heterogeneity was observed between study estimates within both meta-analyses.

The key findings of this meta-analysis strongly support previous research [9,20,22–26]. Within this study, a broad view of victimisation was arrogated, including a range of bullying behaviours such as cyber victimisation, homophobic bullying, peer bullying and so forth. Preceding meta-analyses have previously demonstrated established links between peer victimisation and suicide and LGBT victimisation and non-suicidal self-injury (NSSI) [25,26]. This review demonstrates that there is a high prevalence between LGBTQ+ young people experiencing various forms of victimisation and self-harm and suicide. Indeed, this link between victimisation and self-harm and/or suicide appears to be more common than that among cisgender, heterosexual peers.

Mental health difficulties were also shown to be highly prevalent with self-harm and suicide among LGBTQ+ young people. Liu and colleagues also evidenced mental health difficulties were linked to NSSI within this population [25]. The current review extends findings from previous research by calculating risk prevalence and odds across the spectrum of self-harm to suicide and differentiating by gender identity and sexuality [25,26]. Thus, demonstrating that higher rates of victimisation and mental health difficulties are found in LGBTQ+ young people who experience self-harm and suicide. However, evidence is not available from this review as the causal pathway causing self-harm or suicide or how predictive these risks associated with self-harm and suicide are.

By looking across the broad umbrella LGBTQ+ identities, this review has assessed the prevalence of risks associated with self-harm and suicide by gender identity compared to sexual orientation minorities groups. This allows for consideration of how influential these risks might be to particular groups among the LGBTQ+ label, and where differences of risk may lie. Both victimisation and mental health difficulties were evidenced to be more prevalent within LGBQ young people rather than TGNC. However, it is likely that our finding is due to the higher number of studies focusing solely on LGBQ populations, as noted by the wider confidence intervals seen within the TGNC subgroup analyses. Furthermore, those studies which considered both sexual orientation and gender identity, tend to have low numbers of TGNC participants. Therefore, the TGNC risks are potentially conflated or ignored, as there is a lack of statistical power to evidence risks which may apply to TGNC participants and not LGBQ.

Further to this, we were unable to conduct meta-analysis by identity (e.g. transgender man, transgender woman, nonbinary etc.) within gender identity or sexuality (e.g. bisexual, homosexual, lesbian), thereby these are broadly categorised. This may overlook differences between identifying as a particular sexual orientation or gender identity; and, how being a member of these subgroups may differ from each other [145]. Additionally, no papers considered sexualities outside of homosexual, bisexual, queer or questioning. This limits how far these risk conclusions might be drawn to other sexual orientation groups e.g. those who are asexual, pansexual, polysexual etc. Future research should support inclusion of diverse sexualities and gender identities within studies, offering individuals to self-report in their own words, and options for intersectional identities.

This review has important clinical and policy implications in relation to suicide prevention among LGBTQ young people. Primarily, discrimination against LGBTQ+ individuals has widely been recognised as a priority for governments and organisations globally [146,147]. These results definitively highlight the harmful outcomes associated with acts of discrimination and victimisation. Given the variety of countries which are included in this study, the findings of this study could be used to inform national policies, such that there is a priority focus on reducing minority victimisation and discrimination. Furthermore, by understanding these complex experiences which surround LGBTQ+ youth, compounded by high rates of mental health difficulties, suicide prevention strategies are better informed to support LGBTQ+ youth. Thereby suicide prevention interventions and policies may be better tailored to the specific needs of LGBTQ+ young people and develop initiatives which build resilience and challenge societal acceptance of such discrimination. However, the studies in this meta-analysis mainly come from High-Income Countries (HIC), therefore the results might not be generalisable to Low- and Middle- Income Countries (LMIC) where young people who identify as LGBTQ+ may face additional or different types of risks.

Secondly, health care professionals should be aware of the high prevalence of mental health difficulties and victimisation within the umbrella of LGBTQ+ young people. Acknowledging sexuality and gender identity in an accepting and supportive manner, would be beneficial to encouraging a constructive health care environment [148,149], which could potentially aid disclosure of self-harm and suicide. Evidence also shows that health professionals encouraging LGBTQ+ youth to discuss their experiences of victimisation could further reduce negative health consequences [150]. From these insights, professionals might be able to suggest treatments or care understanding the sociodemographic environment which these individuals are living in.

Much of this research takes places within school settings, with the average age of participants being below 18 years old. Given that bullying among school-aged children is common [151], this would suggest that school-based interventions would be an appropriate setting to target victimisation for LGBTQ+ young people, potentially reducing self-harm and suicide. This is supported by a recent study suggesting that addressing the barriers and facilitators when reporting and responding to LGBTQ+ victimisation in schools would prevent adverse mental health [152]. In particular, LGBTQ+ youth felt that building trust with staff members, being given time to discuss problems and receiving responses from school were key [152]. Therefore, creating an environment which recognises the unique aspects and potential risks of being LGBTQ+, such as dealing with difficult disclosure [118] or understanding gender nonconformity [25] would be beneficial. This could translate to older adolescents and young adults by having similar environments within colleges, universities or social community spaces. These spaces might be able to consider risks, which differentiate by age (e.g. identity development, transition treatments available, housing situations) which due to limited reporting we were unable to meta-analysis within this review.

There is a wealth of literature readily available relating to risks for self-harm and suicide within LGBTQ+ young people. However importantly, even though many of these studies had explicit focus on LGBTQ+ individuals, only 12% of the total population held these identities and reporting is highly inconsistence between individual risks. Future research in the field of self-harm and suicide prevention requires a specific LGBTQ+ focus as this would allow for a holistic understanding of these populations’ experiences.

### Strengths & limitations

This is the first systematic review and meta-analysis which has comprehensively synthesised existing evidence from across the full spectrum of LGBTQ+ young people in order to identify the prevalence of key risks with self-harm and suicide. Firstly, this dimensional approach allowed for a holistic view and comparison of risk prevalence across self-harm and suicidal thoughts and behaviours. Secondly, broad search strategies were run, which ensured a large amount of studies was identified across disciplines and across the LGBTQ+ umbrella. This search was re-run prior to submission to ensure that the review was as up-to-date as possible. Thereby, TGNC populations were able to be identified and specifically examined with reference to similar LGBQ samples. A final strength was the robust meta-analytic strategy which was emplaced within this study, therefore allowing authors to determine points of bias and control for these.

There were, however, some limitations which need to be considered. Firstly, there were few high-quality studies and substantial heterogeneity within the findings. Sources of heterogeneity were explored using our pre-specified subgroup analysis but also to determine points of heterogeneity; this offered little. Potentially, this was related to the use of four variations of the NOS assessment (see SM2). While inclusion of four versions allowed for a greater number of papers to be assessed, this also created another variable of ambiguity. However, heterogeneity may also be related to the variation in conceptualisation of phenomena, population, study design and fundamentally individual reporting of risk. In future, clear operationalisation within studies is necessary and use of standardised, validated measures to assess self-harm and suicide across the spectrum of thoughts and behaviours.

Secondly, self-harm with suicide intention and self-harm without suicide intention may have different associated risks which link to why someone might be more likely to consider suicide. However, given the measures used to assess self-harm within included studies this was not possible. Therefore, only risks associated with self-harm regardless of intention was able to be analysed. This does not allow us to offer explanation as to why someone might consider suicide with this behaviour. Finally, searches were limited to English language; thereby key studies within other languages may have been overlooked.

## Supporting information

S1 Fig Search strategy terms. (TIF)

S2 Fig PRISMA flow diagram. (TIF)

S3 Fig Forest plot of victimisation prevalence among LGBTQ+ with experiences of self-harm or suicide. (TIF)

S4 Fig Odds ratio among LGBTQ+ young people with experiences of self-harm or suicide compared to cisgender, heterosexual young people with experiences of self-harm or suicide. (TIF)

S5 Fig Overall prevalence of mental health difficulties within LGBTQ+ young people with experiences of self-harm or suicide. (TIF)

S6 Fig Odds ratio of LGBTQ+ young people with experiences of self-harm or suicide compared to cisgender, heterosexual peers with experiences of self-harm or suicide. (TIF)

S1 Table Inclusion criteria used during screening process. (DOCX)

S2 Table Risks associated with experiences of self-harm or suicide among LGBTQ+ young people: Data unable to be numerically synthesised. (DOCX)

S3 Table Subgroup analyses of victimisation prevalence among LGBTQ+ young people with self-harm or suicidal experiences. (DOCX)

S4 Table Subgroup analyses of mental health difficulties prevalence among LGBTQ+ populations who have experiences of self-harm or suicide. (DOCX)

S1 File PRISMA checklist. (DOC)

S2 File NOS. (PDF)

S3 File Supplementary results. (DOCX)

## References

[1] World Health Organisation. Suicide across the world. World Health Organisation. 2016.

[2] World Health Organization. Preventing suicide: A global imperative. 2014. Available from: http://www.who.int/mental_health/suicide-prevention/world_report_2014/en/.

[3] Marshal MP, Dietz LJ, Friedman MS, Stall R, Smith HA, McGinley J, et al Suicidality and depression disparities between sexual minority and heterosexual youth: A meta-analytic review. Journal of adolescent health. 2011 8 1;49(2):115–23. 10.1016/j.jadohealth.2011.02.005 21783042 PMC3649127

[4] Surace T, Fusar-Poli L, Vozza L, Cavone V, Arcidiacono C, Mammano R, et al Lifetime prevalence of suicidal ideation and suicidal behaviors in gender non-conforming youths: a meta-analysis. European Child & Adolescent Psychiatry. 2020 3 13:1–5. 10.1007/s00787-020-01508-5 32170434

[5] National Institute for Health and Clinical Excellence. Self-harm: Longer-term management. National Institute for Health and Clinical Excellence; 2011.

[6] Hawton K, Harriss L. Deliberate self-harm in young people: characteristics and subsequent mortality in a 20-year cohort of patients presenting to hospital. The Journal of clinical psychiatry. 2007 10. 17960975

[7] Hawton K, Saunders KE, O'Connor RC. Self-harm and suicide in adolescents. The Lancet. 2012 6 23;379(9834):2373–82. 10.1016/S0140-6736(12)60322-5 22726518

[8] Jourian TJ. Evolving nature of sexual orientation and gender identity. New Directions for Student Services. 2015 12;2015(152):11–23.

[9] Taylor PJ, Dhingra K, Dickson JM, McDermott E. Psychological correlates of self-harm within gay, lesbian and bisexual UK university students. Archives of suicide research. 2018 11 19 10.1080/13811118.2018.1515136 30152727

[10] Clark TC, Lucassen MF, Bullen P, Denny SJ, Fleming TM, Robinson EM, et al The health and well-being of transgender high school students: results from the New Zealand adolescent health survey (Youth'12). Journal of Adolescent Health. 2014 7 1;55(1):93–9. 10.1016/j.jadohealth.2013.11.008 24438852

[11] Fliege H, Lee JR, Grimm A, Klapp BF. Risk factors and correlates of deliberate self-harm behavior: A systematic review. Journal of psychosomatic research. 2009 6 1;66(6):477–93. 10.1016/j.jpsychores.2008.10.013 19446707

[12] Nock MK, Borges G, Bromet EJ, Alonso J, Angermeyer M, Beautrais A, et al Cross-national prevalence and risk factors for suicidal ideation, plans and attempts. The British Journal of Psychiatry. 2008 2;192(2):98–105. 10.1192/bjp.bp.107.040113 18245022 PMC2259024

[13] Plener PL, Kaess M, Schmahl C, Pollak S, Fegert JM, Brown RC. Nonsuicidal self-injury in adolescents. Deutsches Ärzteblatt international. 2018 1;115(3):23 10.3238/arztebl.2018.0023 29366448 PMC5787659

[14] Carballo JJ, Llorente C, Kehrmann L, Flamarique I, Zuddas A, Purper-Ouakil D, et al Psychosocial risk factors for suicidality in children and adolescents. European Child & Adolescent Psychiatry. 2019 1 25:1–8. 10.1007/s00787-018-01270-9 30684089 PMC7305074

[15] Clarke S, Allerhand LA, Berk MS. Recent advances in understanding and managing self-harm in adolescents. F1000Research. 2019;8 10.12688/f1000research.17047.1 31681470 PMC6816451

[16] Boyas JF, Villarreal-Otálora T, Alvarez-Hernandez LR, Fatehi M. Suicide ideation, planning, and attempts: the case of the Latinx LGB youth. Health promotion perspectives. 2019;9(3):198 10.15171/hpp.2019.28 31508340 PMC6717921

[17] Consolacion TB, Russell ST, Sue S. Sex, race/ethnicity, and romantic attractions: Multiple minority status adolescents and mental health. Cultural Diversity and Ethnic Minority Psychology. 2004 8;10(3):200 10.1037/1099-9809.10.3.200 15311974 PMC3559128

[18] Bostwick WB, Meyer I, Aranda F, Russell S, Hughes T, Birkett M, et al Mental health and suicidality among racially/ethnically diverse sexual minority youths. American journal of public health. 2014 6;104(6):1129–36. 10.2105/AJPH.2013.301749 24825217 PMC4062032

[19] Wang PW, Ko NY, Hsiao RC, Chen MH, Lin HC, Yen CF. Suicidality among gay and bisexual men in Taiwan: Its relationships with sexuality and gender role characteristics, homophobic bullying victimization, and social support. Suicide and Life‐Threatening Behavior. 2019 4;49(2):466–77. 10.1111/sltb.12451 29520832

[20] Meyer IH. Minority stress and mental health in gay men. Journal of health and social behavior. 1995 3 1:38–56. 7738327

[21] Grossman AH, D'augelli AR, Frank JA. Aspects of psychological resilience among transgender youth. Journal of LGBT youth. 2011 3 29;8(2):103–15.

[22] Brandelli Costa A, Pasley A, Machado WD, Alvarado E, Dutra-Thomé L, Koller SH. The experience of sexual stigma and the increased risk of attempted suicide in young Brazilian people from low socioeconomic group. Frontiers in psychology. 2017 2 22;8:192 10.3389/fpsyg.2017.00192 28275356 PMC5319990

[23] Bailey L, Ellis SJ, McNeil J. Suicide risk in the UK trans population and the role of gender transition in decreasing suicidal ideation and suicide attempt. The Mental Health Review. 2014 Dec 1;19(4):209.

[24] Remafedi G, Farrow JA, Deisher RW. Risk factors for attempted suicide in gay and bisexual youth. Pediatrics. 1991 6 1;87(6):869–75. 2034492

[25] Liu RT, Sheehan AE, Walsh RF, Sanzari CM, Cheek SM, Hernandez EM. Prevalence and correlates of non-suicidal self-injury among lesbian, gay, bisexual, and transgender individuals: A systematic review and meta-analysis. Clinical psychology review. 2019 12 1;74:101783 10.1016/j.cpr.2019.101783 31734440 PMC6896220

[26] Hatchel T, Polanin JR, Espelage DL. Suicidal thoughts and behaviors among LGBTQ youth: meta-analyses and a systematic review. Archives of suicide research. 2019 9 13:1–37. 10.1080/13811118.2019.1663329 31597541

[27] Orlando CM, Broman-Fulks JJ, Whitlock JL, Curtin L, Michael KD. Nonsuicidal self-injury and suicidal self-injury: A taxometric investigation. Behavior therapy. 2015 11 1;46(6):824–33. 10.1016/j.beth.2015.01.002 26520224

[28] King M, Semlyen J, Tai SS, Killaspy H, Osborn D, Popelyuk D, et al A systematic review of mental disorder, suicide, and deliberate self harm in lesbian, gay and bisexual people. BMC psychiatry. 2008 12 1;8(1):70. 18706118 10.1186/1471-244X-8-70 PMC2533652

[29] Shamseer L, Moher D, Clarke M, Ghersi D, Liberati A, Petticrew M, et al Preferred reporting items for systematic review and meta-analysis protocols (PRISMA-P) 2015: elaboration and explanation. Bmj. 2015 1 2;349 10.1136/bmj.g7647 25555855

[30] Williams AJ, Arcelus J, Townsend E, Michail M. Examining risk factors for self-harm and suicide in LGBTQ+ young people: a systematic review protocol. BMJ open. 2019 11 1;9(11). 10.1136/bmjopen-2019-031541 31719085 PMC6858144

[31] Arnett JJ, Hughes M. Adolescence and emerging adulthood. Boston, MA: Pearson; 2014 10.1177/2167696814561999

[32] Burt BA. Definitions of risk. Journal of dental education. 2001 10;65(10):1007–8. 11699970

[33] Byrt T, Bishop J, Carlin JB. Bias, prevalence and kappa. Journal of clinical epidemiology. 1993 5 1;46(5):423–9. 10.1016/0895-4356(93)90018-v 8501467

[34] Chen G, Faris P, Hemmelgarn B, Walker RL, Quan H. Measuring agreement of administrative data with chart data using prevalence unadjusted and adjusted kappa. BMC medical research methodology. 2009 12 1;9(1):5 10.1186/1471-2288-9-5 19159474 PMC2636838

[35] Knipe D, Williams AJ, Hannam-Swain S, Upton S, Brown K, Bandara P, et al Psychiatric morbidity and suicidal behaviour in low-and middle-income countries: A systematic review and meta-analysis. PLoS medicine. 2019 10 9;16(10):e1002905 10.1371/journal.pmed.1002905 31597983 PMC6785653

[36] Borenstein M, Cooper H, Hedges L, Valentine J. Effect sizes for continuous data. The handbook of research synthesis and meta-analysis. 2009 2 5;2:221–35.

[37] Wells GA, Shea BJ. O&apos; Connell D, Peterson J, Welch V, Losos M, et al The Newcastle–Ottawa Scale (NOS) for Assessing the Quality of Non-Randomized Studies in Meta-Analysis. Applied Engineering in Agriculture. 2014;18(6):727–34.

[38] Herzog R, Álvarez-Pasquin MJ, Díaz C, Del Barrio JL, Estrada JM, Gil Á. Are healthcare workers’ intentions to vaccinate related to their knowledge, beliefs and attitudes? A systematic review. BMC public health. 2013 12 1;13(1):154 10.1186/1471-2458-13-154 23421987 PMC3602084

[39] Polihronis C, Cloutier P, Kaur J, Skinner R, Cappelli M. What’s the harm in asking? A systematic review and meta-analysis on the risks of asking about suicide-related behaviors and self-harm with quality appraisal. Archives of Suicide Research. 2020 7 23:1–23. 10.1080/13811118.2020.1793857 32715986

[40] Mars B, Heron J, Klonsky ED, Moran P, O'Connor RC, Tilling K, et al Predictors of future suicide attempt among adolescents with suicidal thoughts or non-suicidal self-harm: a population-based birth cohort study. The Lancet Psychiatry. 2019 4 1;6(4):327–37. 10.1016/S2215-0366(19)30030-6 30879972 PMC6494973

[41] Baams L, Grossman AH, Russell ST. Minority stress and mechanisms of risk for depression and suicidal ideation among lesbian, gay, and bisexual youth. Developmental Psychology. 2015 5;51(5):688 10.1037/a0038994 25751098 PMC4412799

[42] D'augelli AR, Hershberger SL. Lesbian, gay, and bisexual youth in community settings: Personal challenges and mental health problems. American journal of community psychology. 1993 8;21(4):421–48. 10.1007/BF00942151 8192119

[43] Mendoza-Pérez JC, Ortiz-Hernández L. Violence as mediating variable in mental health disparities associated to sexual orientation among Mexican youths. Journal of homosexuality. 2019 3 21;66(4):510–32. 10.1080/00918369.2017.1422938 29303429

[44] DerSimonian R, Laird N. Meta-analysis in clinical trials. Controlled clinical trials. 1986 9 1;7(3):177–88. 10.1016/0197-2456(86)90046-2 3802833

[45] Orwin RG. A fail-safe N for effect size in meta-analysis. Journal of educational statistics. 1983 6;8(2):157–9.

[46] Hershberger SL, Pilkington NW, D'Augelli AR. Predictors of suicide attempts among gay, lesbian, and bisexual youth. Journal of Adolescent Research. 1997 10;12(4):477–97.

[47] Huang Y, Li P, Lai Z, Jia X, Xiao D, Wang T, et al Association between sexual minority status and suicidal behavior among Chinese adolescents: A moderated mediation model. Journal of affective disorders. 2018 10 15;239:85–92. 10.1016/j.jad.2018.07.004 29990667

[48] D'augelli AR, Hershberger SL. Lesbian, gay, and bisexual youth in community settings: Personal challenges and mental health problems. American journal of community psychology. 1993 8;21(4):421–48. 10.1007/BF00942151 8192119

[49] Huang Y, Li P, Lai Z, Jia X, Xiao D, Wang T, et al Role of sleep quality in mediating the relationship between sexual minority status and suicidal behavior among Chinese adolescents. Psychology research and behavior management. 2018;11:607 10.2147/PRBM.S186586 30573996 PMC6292244

[50] Antonio R, Moleiro C. Social and parental support as moderators of the effects of homophobic bullying on psychological distress in youth. Psychology in the Schools. 2015 9;52(8):729–42.

[51] Baiden P, Mengo C, Small E. History of physical teen dating violence and its association with suicidal behaviors among adolescent high school students: results from the 2015 Youth Risk Behavior Survey. Journal of interpersonal violence. 2019 7 4:0886260519860087 10.1177/0886260519860087 31271096

[52] Ballard ME, Jameson JP, Martz DM. Sexual identity and risk behaviors among adolescents in rural Appalachia. Journal of Rural Mental Health. 2017 1;41(1):17.

[53] Birkett M, Espelage DL, Koenig B. LGB and questioning students in schools: The moderating effects of homophobic bullying and school climate on negative outcomes. Journal of youth and adolescence. 2009 8 1;38(7):989–1000. 10.1007/s10964-008-9389-1 19636741 PMC6322390

[54] Burton CM, Marshal MP, Chisolm DJ, Sucato GS, Friedman MS. Sexual minority-related victimization as a mediator of mental health disparities in sexual minority youth: A longitudinal analysis. Journal of youth and adolescence. 2013 3 1;42(3):394–402. 10.1007/s10964-012-9901-5 23292751 PMC3570607

[55] Button DM. Understanding the effects of victimization: Applying general strain theory to the experiences of LGBQ youth. Deviant Behavior. 2016 5 3;37(5):537–56.

[56] Cénat JM, Blais M, Hébert M, Lavoie F, Guerrier M. Correlates of bullying in Quebec high school students: The vulnerability of sexual-minority youth. Journal of affective disorders. 2015 9 1;183:315–21. 10.1016/j.jad.2015.05.011 26047959 PMC4641744

[57] Coulter RW, Blosnich JR, Bukowski LA, Herrick AL, Siconolfi DE, Stall RD. Differences in alcohol use and alcohol-related problems between transgender-and nontransgender-identified young adults. Drug and alcohol dependence. 2015 9 1;154:251–9. 10.1016/j.drugalcdep.2015.07.006 26210734 PMC4536098

[58] Cutuli JJ, Treglia D, Herbers JE. Adolescent homelessness and associated features: prevalence and risk across eight states. Child Psychiatry & Human Development. 2020 2 1;51(1):48–58. 10.1007/s10578-019-00909-1 31270658

[59] D'Augelli AR, Hershberger SL, Pilkington NW. Suicidality patterns and sexual orientation-related factors among lesbian, gay, and bisexual youths. Suicide and life-threatening behavior. 2001 9 1;31(3):250–64. 10.1521/suli.31.3.250.24246 11577911

[60] D’Augelli AR, Grossman AH, Salter NP, Vasey JJ, Starks MT, Sinclair KO. Predicting the suicide attempts of lesbian, gay, and bisexual youth. Suicide and life-threatening behavior. 2005 12 1;35(6):646–60. 10.1521/suli.2005.35.6.646 16552980

[61] Assis SG, Gomes R, Pires TD. Adolescence, sexual behavior and risk factors to health. Revista de saude publica. 2014;48:43–51. 10.1590/s0034-8910.2014048004638 24789636 PMC4206123

[62] Duncan DT, Hatzenbuehler ML. Lesbian, gay, bisexual, and transgender hate crimes and suicidality among a population-based sample of sexual-minority adolescents in Boston. American journal of public health. 2014 2;104(2):272–8. 10.2105/AJPH.2013.301424 24328619 PMC3935714

[63] DuRant RH, Krowchuk DP, Sinal SH. Victimization, use of violence, and drug use at school among male adolescents who engage in same-sex sexual behavior. The Journal of pediatrics. 1998 7 1;133(1):113–8. 10.1016/s0022-3476(98)70189-1 9672522

[64] Eisenberg ME, McMorris BJ, Gower AL, Chatterjee D. Bullying victimization and emotional distress: is there strength in numbers for vulnerable youth?. Journal of psychosomatic research. 2016 7 1;86:13–9. 10.1016/j.jpsychores.2016.04.007 27302541

[65] Eisenberg ME, Gower AL, McMorris BJ, Rider GN, Coleman E. Emotional distress, bullying victimization, and protective factors among transgender and gender diverse adolescents in city, suburban, town, and rural locations. The Journal of Rural Health. 2019 3;35(2):270–81. 10.1111/jrh.12311 29940070 PMC6309763

[66] Espelage DL, Aragon SR, Birkett M, Koenig BW. Homophobic teasing, psychological outcomes, and sexual orientation among high school students: What influence do parents and schools have?. School psychology review. 2008 6 1;37(2):202–16.

[67] Espelage DL, Merrin GJ, Hatchel T. Peer victimization and dating violence among LGBTQ youth: The impact of school violence and crime on mental health outcomes. Youth violence and juvenile justice. 2018 4;16(2):156–73.

[68] Fraser G, Wilson MS, Garisch JA, Robinson K, Brocklesby M, Kingi T, et al Non-suicidal self-injury, sexuality concerns, and emotion regulation among sexually diverse adolescents: A multiple mediation analysis. Archives of suicide research. 2018 7 3;22(3):432–52. 10.1080/13811118.2017.1358224 28759324

[69] Friedman MS, Koeske GF, Silvestre AJ, Korr WS, Sites EW. The impact of gender-role nonconforming behavior, bullying, and social support on suicidality among gay male youth. Journal of Adolescent Health. 2006 5 1;38(5):621–3. 10.1016/j.jadohealth.2005.04.014 16635780

[70] Garofalo R, Wolf RC, Wissow LS, Woods ER, Goodman E. Sexual orientation and risk of suicide attempts among a representative sample of youth. Archives of pediatrics & adolescent medicine. 1999 5 1;153(5):487–93. 10.1001/archpedi.153.5.487 10323629

[71] Gibbs JJ, Goldbach J. Religious conflict, sexual identity, and suicidal behaviors among LGBT young adults. Archives of Suicide Research. 2015 10 2;19(4):472–88. 10.1080/13811118.2015.1004476 25763926 PMC4706071

[72] Grossman AH, Kerner MS. Self-esteem and supportiveness as predictors of emotional distress in gay male and lesbian youth. Journal of Homosexuality. 1998 2 13;35(2):25–39. 10.1300/J082v35n02_02 9524920

[73] Grossman AH, D'Augelli AR. Transgender youth and life-threatening behaviors. Suicide and life-threatening behavior. 2007 10;37(5):527–37. 10.1521/suli.2007.37.5.527 17967119

[74] Grossman AH, Park JY, Russell ST. Transgender youth and suicidal behaviors: Applying the interpersonal psychological theory of suicide. Journal of gay & lesbian mental health. 2016 10 1;20(4):329–49. 10.1080/19359705.2016.1207581 28344728 PMC5363722

[75] Halkitis PN, Cook SH, Ristuccia A, Despotoulis J, Levy MD, Bates FC, et al Psychometric analysis of the Life Worries Scale for a new generation of sexual minority men: The P18 Cohort Study. Health Psychology. 2018 1;37(1):89 10.1037/hea0000537 28967772 PMC5764782

[76] Kim GH, Ahn HS, Kim HJ. Type of sexual intercourse experience and suicidal ideation, plans, and attempts among youths: a cross-sectional study in South Korea. BMC Public Health. 2016 12 1;16(1):1229 10.1186/s12889-016-3895-y 27923371 PMC5142152

[77] Tejera CH, Horner-Johnson W, Andresen EM. Application of an intersectional framework to understanding the association of disability and sexual orientation with suicidal ideation among Oregon Teens. Disability and health journal. 2019 10 1;12(4):557–63. 10.1016/j.dhjo.2019.05.006 31167741

[78] Hightow-Weidman LB, Phillips G, Jones KC, Outlaw AY, Fields SD, Smith, for The YMSM of Color SPNS Initiative Study Group JC. Racial and sexual identity-related maltreatment among minority YMSM: prevalence, perceptions, and the association with emotional distress. AIDS patient care and STDs. 2011 8 1;25(S1):S39–45. 21688988 10.1089/apc.2011.9877

[79] Huang Y, Li P, Lai Z, Jia X, Xiao D, Wang T, et al Chinese Sexual Minority Male Adolescents’ Suicidality and Body Mass Index. International journal of environmental research and public health. 2018 11;15(11):2558 10.3390/ijerph15112558 30445669 PMC6266787

[80] King MT, Merrin GJ, Espelage DL, Grant NJ, Bub KL. Suicidality and intersectionality among students identifying as nonheterosexual and with a disability. Exceptional Children. 2018 Jan;84(2):141–58.

[81] Lardier DT Jr, Bermea AM, Pinto SA, Garcia-Reid P, Reid RJ. The relationship between sexual minority status and suicidal ideations among urban Hispanic adolescents. Journal of LGBT issues in counseling. 2017 7 3;11(3):174–89.

[82] LeVasseur MT, Kelvin EA, Grosskopf NA. Intersecting identities and the association between bullying and suicide attempt among New York city youths: results from the 2009 New York city youth risk behavior survey. American journal of public health. 2013 6;103(6):1082–9. 10.2105/AJPH.2012.300994 23597376 PMC3698714

[83] Li X, Zheng H, Tucker W, Xu W, Wen X, Lin Y, et al Research on relationships between sexual identity, adverse childhood experiences and non-suicidal self-injury among rural high school students in less developed areas of China. International journal of environmental research and public health. 2019 1;16(17):3158 10.3390/ijerph16173158 31470648 PMC6747706

[84] Liu RT, Mustanski B. Suicidal ideation and self-harm in lesbian, gay, bisexual, and transgender youth. American journal of preventive medicine. 2012 3 1;42(3):221–8. 10.1016/j.amepre.2011.10.023 22341158

[85] Lytle MC, Silenzio VM, Homan CM, Schneider P, Caine ED. Suicidal and help-seeking behaviors among youth in an online lesbian, gay, bisexual, transgender, queer, and questioning social network. Journal of homosexuality. 2018 11 10;65(13):1916–33. 10.1080/00918369.2017.1391552 29020574

[86] Marx RA, Hatchel T, Mehring CB, Espelage DL. Predictors of sexual victimisation and suicidal ideation among transgender and gender-nonconforming adolescents. Psychology & Sexuality. 2019 Nov 21:1–7.

[87] Mustanski BS, Garofalo R, Emerson EM. Mental health disorders, psychological distress, and suicidality in a diverse sample of lesbian, gay, bisexual, and transgender youths. American journal of public health. 2010 12;100(12):2426–32. 10.2105/AJPH.2009.178319 20966378 PMC2978194

[88] Mustanski B, Andrews R, Herrick A, Stall R, Schnarrs PW. A syndemic of psychosocial health disparities and associations with risk for attempting suicide among young sexual minority men. American journal of public health. 2014 2;104(2):287–94. 10.2105/AJPH.2013.301744 24328641 PMC3935701

[89] Palm A, Danielsson I, Skalkidou A, Olofsson N, Högberg U. Violence victimisation—a watershed for young women’s mental and physical health. The European Journal of Public Health. 2016 10 1;26(5):861–7. 10.1093/eurpub/ckv234 26743590 PMC5054268

[90] Poteat VP, Aragon SR, Espelage DL, Koenig BW. Psychosocial concerns of sexual minority youth: Complexity and caution in group differences. Journal of consulting and clinical psychology. 2009 2;77(1):196 10.1037/a0014158 19170465

[91] Proctor CD, Groze VK. Risk factors for suicide among gay, lesbian, and bisexual youths. Social work. 1994 9 1;39(5):504–13. 10.1093/sw/39.5.504 7939864

[92] Puckett JA, Horne SG, Surace F, Carter A, Noffsinger-Frazier N, Shulman J, et al Predictors of sexual minority youth’s reported suicide attempts and mental health. Journal of homosexuality. 2017 5 12;64(6):697–715. 10.1080/00918369.2016.1196999 27268386

[93] Remafedi G. Suicidality in a venue-based sample of young men who have sex with men. Journal of adolescent health. 2002 10 1;31(4):305–10. 10.1016/s1054-139x(02)00405-6 12359375

[94] Rimes KA, Goodship N, Ussher G, Baker D, West E. Non-binary and binary transgender youth: Comparison of mental health, self-harm, suicidality, substance use and victimization experiences. International Journal of Transgenderism. 2019 7 3;20(2–3):230–40. 10.1080/15532739.2017.1370627 32999609 PMC6831005

[95] Rotheram-Borus MJ, Hunter J, Rosario M. Suicidal behavior and gay-related stress among gay and bisexual male adolescents. Journal of adolescent research. 1994 10;9(4):498–508.

[96] Russell ST, Joyner K. Adolescent sexual orientation and suicide risk: Evidence from a national study. American Journal of public health. 2001 8;91(8):1276–81. 10.2105/ajph.91.8.1276 11499118 PMC1446760

[97] Ryan C, Huebner D, Diaz RM, Sanchez J. Family rejection as a predictor of negative health outcomes in white and Latino lesbian, gay, and bisexual young adults. Pediatrics. 2009 1 1;123(1):346–52. 10.1542/peds.2007-3524 19117902

[98] Savin-Williams RC, Ream GL. Suicide attempts among sexual-minority male youth. Journal of Clinical Child and Adolescent Psychology. 2003 11 1;32(4):509–22. 10.1207/S15374424JCCP3204_3 14710459

[99] Scheer JR, McConocha E, Behari K, Pachankis JE. Sexual violence as a mediator of sexual orientation disparities in alcohol use, suicidality, and sexual-risk behaviour among female youth. Psychology & Sexuality. 2019 11 22:1–5. 10.1080/19419899.2019.1690031 PMC8112808 33981383

[100] Shearer A, Russon J, Herres J, Wong A, Jacobs C, Diamond GM, et al Religion, sexual orientation, and suicide attempts among a sample of suicidal adolescents. Suicide and Life‐Threatening Behavior. 2018 8;48(4):431–7. 10.1111/sltb.12372 28726309

[101] Taliaferro LA, Gloppen KM, Muehlenkamp JJ, Eisenberg ME. Depression and suicidality among bisexual youth: A nationally representative sample. Journal of LGBT youth. 2018 1 2;15(1):16–31.

[102] Taliaferro LA, McMorris BJ, Rider GN, Eisenberg ME. Risk and protective factors for self-harm in a population-based sample of transgender youth. Archives of suicide research. 2019 4 3;23(2):203–21. 10.1080/13811118.2018.1430639 29461934 PMC6102088

[103] Teasdale B, Bradley-Engen MS. Adolescent same-sex attraction and mental health: The role of stress and support. Journal of Homosexuality. 2010 1 29;57(2):287–309. 10.1080/00918360903489127 20390994

[104] Thoma BC, Huebner DM. Health consequences of racist and antigay discrimination for multiple minority adolescents. Cultural Diversity and Ethnic Minority Psychology. 2013 10;19(4):404 10.1037/a0031739 23731232 PMC4086429

[105] Waldo CR, Hesson-McInnis MS, D'Augelli AR. Antecedents and consequences of victimization of lesbian, gay, and bisexual young people: A structural model comparing rural university and urban samples. American Journal of Community Psychology. 1998 4 1;26(2):307–34. 10.1023/a:1022184704174 9693694

[106] Walls NE, Freedenthal S, Wisneski H. Suicidal ideation and attempts among sexual minority youths receiving social services. Social work. 2008 1 1;53(1):21–9. 10.1093/sw/53.1.21 18610818

[107] Walls NE, Laser J, Nickels SJ, Wisneski H. Correlates of cutting behavior among sexual minority youths and young adults. Social Work Research. 2010 12 1;34(4):213–26.

[108] Yadegarfard M, Ho R, Bahramabadian F. Influences on loneliness, depression, sexual-risk behaviour and suicidal ideation among Thai transgender youth. Culture, health & sexuality. 2013 6 1;15(6):726–37. 10.1080/13691058.2013.784362 23659441

[109] Yadegarfard M, Meinhold-Bergmann ME, Ho R. Family rejection, social isolation, and loneliness as predictors of negative health outcomes (depression, suicidal ideation, and sexual risk behavior) among Thai male-to-female transgender adolescents. Journal of LGBT Youth. 2014 10 2;11(4):347–63.

[110] Almeida J, Johnson RM, Corliss HL, Molnar BE, Azrael D. Emotional distress among LGBT youth: The influence of perceived discrimination based on sexual orientation. Journal of youth and adolescence. 2009 8 1;38(7):1001–14. 10.1007/s10964-009-9397-9 19636742 PMC3707280

[111] Arcelus J, Claes L, Witcomb GL, Marshall E, Bouman WP. Risk factors for non-suicidal self-injury among trans youth. The Journal of Sexual Medicine. 2016 3 1;13(3):402–12. 10.1016/j.jsxm.2016.01.003 26944465

[112] Berona J, Horwitz AG, Czyz EK, King CA. Predicting suicidal behavior among lesbian, gay, bisexual, and transgender youth receiving psychiatric emergency services. Journal of psychiatric research. 2020 3 1;122:64–9. 10.1016/j.jpsychires.2019.12.007 31927267 PMC7388154

[113] Blosnich J, Bossarte R. Drivers of disparity: Differences in socially based risk factors of self-injurious and suicidal behaviors among sexual minority college students. Journal of American College Health. 2012 2 1;60(2):141–9. 10.1080/07448481.2011.623332 22316411 PMC3340564

[114] Bontempo DE, d’Augelli AR. Effects of at-school victimization and sexual orientation on lesbian, gay, or bisexual youths’ health risk behavior. Journal of Adolescent health. 2002 5 1;30(5):364–74. 10.1016/s1054-139x(01)00415-3 11996785

[115] Butler C, Joiner R, Bradley R, Bowles M, Bowes A, Russell C, et al Self-harm prevalence and ideation in a community sample of cis, trans and other youth. International Journal of Transgenderism. 2019 10 2;20(4):447–58. 10.1080/15532739.2019.1614130 PMC6913646 32999629

[116] Duong J, Bradshaw C. Associations between bullying and engaging in aggressive and suicidal behaviors among sexual minority youth: The moderating role of connectedness. Journal of school health. 2014 10;84(10):636–45. 10.1111/josh.12196 25154527

[117] Feinstein BA, Turner BC, Beach LB, Korpak AK, Phillips G. Racial/ethnic differences in mental health, substance use, and bullying victimization among self-identified bisexual high school-aged youth. LGBT health. 2019 6 1;6(4):174–83. 10.1089/lgbt.2018.0229 31033384 PMC6551981

[118] Gnan GH, Rahman Q, Ussher G, Baker D, West E, Rimes KA. General and LGBTQ-specific factors associated with mental health and suicide risk among LGBTQ students. Journal of Youth Studies. 2019 11 26;22(10):1393–408.

[119] Goldbach JT, Schrager SM, Mamey MR. Criterion and divergent validity of the sexual minority adolescent stress inventory. Frontiers in psychology. 2017 11 28;8:2057 10.3389/fpsyg.2017.02057 29234292 PMC5712417

[120] Goodenow C, Szalacha L, Westheimer K. School support groups, other school factors, and the safety of sexual minority adolescents. Psychology in the Schools. 2006 5;43(5):573–89.

[121] Hatchel T, Ingram KM, Mintz S, Hartley C, Valido A, Espelage DL, et al Predictors of suicidal ideation and attempts among LGBTQ adolescents: The roles of help-seeking beliefs, peer victimization, depressive symptoms, and drug use. Journal of Child and Family Studies. 2019 9 1;28(9):2443–55. 10.1007/s10826-019-01339-2 PMC11452144 39372436

[122] Hatchel T, Merrin GJ, Espelage AD. Peer victimization and suicidality among LGBTQ youth: the roles of school belonging, self-compassion, and parental support. Journal of LGBT Youth. 2019 4 3;16(2):134–56.

[123] Hatzenbuehler ML. The social environment and suicide attempts in lesbian, gay, and bisexual youth. Pediatrics. 2011 5 1;127(5):896–903. 10.1542/peds.2010-3020 21502225 PMC3081186

[124] Hegna K, Wichstrøm L. Suicide attempts among Norwegian gay, lesbian and bisexual youths: General and specific risk factors. Acta Sociologica. 2007 3;50(1):21–37.

[125] Langhinrichsen-Rohling J, Lamis DA, Malone PS. Sexual attraction status and adolescent suicide proneness: The roles of hopelessness, depression, and social support. Journal of Homosexuality. 2010 12 30;58(1):52–82. 10.1080/00918369.2011.533628 21213175

[126] McDermott E, Hughes E, Rawlings V. The social determinants of lesbian, gay, bisexual and transgender youth suicidality in England: a mixed methods study. Journal of Public Health. 2018 9 1;40(3):e244–51. 10.1093/pubmed/fdx135 29045707 PMC6166581

[127] McKay T, Berzofsky M, Landwehr J, Hsieh P, Smith A. Suicide etiology in youth: Differences and similarities by sexual and gender minority status. Children and Youth Services Review. 2019 7 1;102:79–90.

[128] Mustanski B, Liu RT. A longitudinal study of predictors of suicide attempts among lesbian, gay, bisexual, and transgender youth. Archives of sexual behavior. 2013 4 1;42(3):437–48. 10.1007/s10508-012-0013-9 23054258

[129] Peng K, Zhu X, Gillespie A, Wang Y, Gao Y, Xin Y, et al Self-reported rates of abuse, neglect, and bullying experienced by transgender and gender-nonbinary adolescents in China. JAMA network open. 2019 9 4;2(9):e1911058–. 10.1001/jamanetworkopen.2019.11058 31490542 PMC6735403

[130] Perez-Brumer A, Day JK, Russell ST, Hatzenbuehler ML. Prevalence and correlates of suicidal ideation among transgender youth in California: Findings from a representative, population-based sample of high school students. Journal of the American Academy of Child & Adolescent Psychiatry. 2017 9 1;56(9):739–46. 10.1016/j.jaac.2017.06.010 28838578 PMC5695881

[131] Peterson CM, Matthews A, Copps‐Smith E, Conard LA. Suicidality, self‐harm, and body dissatisfaction in transgender adolescents and emerging adults with gender dysphoria. Suicide and Life‐Threatening Behavior. 2017 8;47(4):475–82. 10.1111/sltb.12289 27539381

[132] Reisner SL, Biello K, Perry NS, Gamarel KE, Mimiaga MJ. A compensatory model of risk and resilience applied to adolescent sexual orientation disparities in nonsuicidal self-injury and suicide attempts. American Journal of Orthopsychiatry. 2014 9;84(5):545 10.1037/ort0000008 25089757

[133] Rimes KA, Shivakumar S, Ussher G, Baker D, Rahman Q, West E. Psychosocial factors associated with suicide attempts, ideation, and future risk in lesbian, gay, and bisexual youth. Crisis. 2018 6 21. 10.1027/0227-5910/a000527 29932021

[134] Shields JP, Whitaker K, Glassman J, Franks HM, Howard K. Impact of victimization on risk of suicide among lesbian, gay, and bisexual high school students in San Francisco. Journal of Adolescent Health. 2012 4 1;50(4):418–20. 10.1016/j.jadohealth.2011.07.009 22443849

[135] Smith BC, Armelie AP, Boarts JM, Brazil M, Delahanty DL. PTSD, depression, and substance use in relation to suicidality risk among traumatized minority lesbian, gay, and bisexual youth. Archives of suicide research. 2016 1 2;20(1):80–93. 10.1080/13811118.2015.1004484 26756389

[136] Smith DM, Wang SB, Carter ML, Fox KR, Hooley JM. Longitudinal predictors of self-injurious thoughts and behaviors in sexual and gender minority adolescents. Journal of Abnormal Psychology. 2020 1;129(1):114 10.1037/abn0000483 31657599

[137] Taliaferro LA, Muehlenkamp JJ. Nonsuicidal self-injury and suicidality among sexual minority youth: risk factors and protective connectedness factors. Academic pediatrics. 2017 9 1;17(7):715–22. 10.1016/j.acap.2016.11.002 28865597

[138] Taliaferro LA, McMorris BJ, Eisenberg ME. Connections that moderate risk of non-suicidal self-injury among transgender and gender non-conforming youth. Psychiatry research. 2018 10 1;268:65–7. 10.1016/j.psychres.2018.06.068 30005190 PMC6129209

[139] Turpin R, Boekeloo B, Dyer T. Sexual identity modifies the association between bullying and suicide planning among adolescents with same-sex sexual partners. Journal of LGBT Youth. 2019 7 3;16(3):300–16.

[140] Veale JF, Peter T, Travers R, Saewyc EM. Enacted stigma, mental health, and protective factors among transgender youth in Canada. Transgender Health. 2017 12 1;2(1):207–16. 10.1089/trgh.2017.0031 29279875 PMC5734137

[141] Wang PW, Ko NY, Hsiao RC, Chen MH, Lin HC, Yen CF. Suicidality among gay and bisexual men in Taiwan: Its relationships with sexuality and gender role characteristics, homophobic bullying victimization, and social support. Suicide and Life‐Threatening Behavior. 2019 4;49(2):466–77. 10.1111/sltb.12451 29520832

[142] Whitaker K, Shapiro VB, Shields JP. School-based protective factors related to suicide for lesbian, gay, and bisexual adolescents. Journal of Adolescent Health. 2016 1 1;58(1):63–8. 10.1016/j.jadohealth.2015.09.008 26707230

[143] Wilson EC, Chen YH, Arayasirikul S, Raymond HF, McFarland W. The impact of discrimination on the mental health of trans* female youth and the protective effect of parental support. AIDS and Behavior. 2016 10 1;20(10):2203–11. 10.1007/s10461-016-1409-7 27115401 PMC5025345

[144] Ybarra ML, Mitchell KJ, Kosciw JG, Korchmaros JD. Understanding linkages between bullying and suicidal ideation in a national sample of LGB and heterosexual youth in the United States. Prevention Science. 2015 4 1;16(3):451–62. 10.1007/s11121-014-0510-2 25322949

[145] Mink MD, Lindley LL, Weinstein AA. Stress, stigma, and sexual minority status: The intersectional ecology model of LGBTQ health. Journal of Gay & Lesbian Social Services. 2014 10 2;26(4):502–21.

[146] Government Equalities Office. LGBT action plan: Improving the Lives of lesbian, gay, bisexual and transgender people. 2018.

[147] The Equilty Project. Australian LGBTQIA+ Policy Guide 2020 [Internet]. 2020. Available from: www.equality.org.au/policy.

[148] Banerjee SC, Staley JM, Alexander K, Walters CB, Parker PA. Encouraging patients to disclose their lesbian, gay, bisexual, or transgender (LGBT) status: oncology health care providers’ perspectives. Translational behavioral medicine. 2020 8;10(4):918–27. 10.1093/tbm/iby105 30476333 PMC7543074

[149] Makadon HJ. Ending LGBT invisibility in health care: The first step in ensuring equitable care. Cleve Clin J Med. 2011 4 1;78(4):220–4. 10.3949/ccjm.78gr.10006 21460126

[150] Earnshaw VA, Reisner SL, Juvonen J, Hatzenbuehler ML, Perrotti J, Schuster MA. LGBTQ bullying: translating research to action in pediatrics. Pediatrics. 2017 10 1;140(4). 10.1542/peds.2017-0432 PMC5613818 28947607

[151] Swearer SM, Espelage DL, Vaillancourt T, Hymel S. What can be done about school bullying? Linking research to educational practice. Educational researcher. 2010 1;39(1):38–47.

[152] Reisner SL, Sava LM, Menino DD, Perrotti J, Barnes TN, Humphrey DL, et al Addressing LGBTQ student bullying in Massachusetts schools: perspectives of LGBTQ students and school health professionals. Prevention science. 2020 4;21(3):408–21. 10.1007/s11121-019-01084-4 31933159 PMC11987988

