# Supplementary figures and images for: A systematic review and meta-analysis of victimisation and mental health prevalence among LGBTQ+ young people with experiences of self-harm and suicide
Source: PLoS One. 2021 Jan 22;16(1):e0245268. doi: 10.1371/journal.pone.0245268 (PMC7822285; doi:10.1371/journal.pone.0245268)

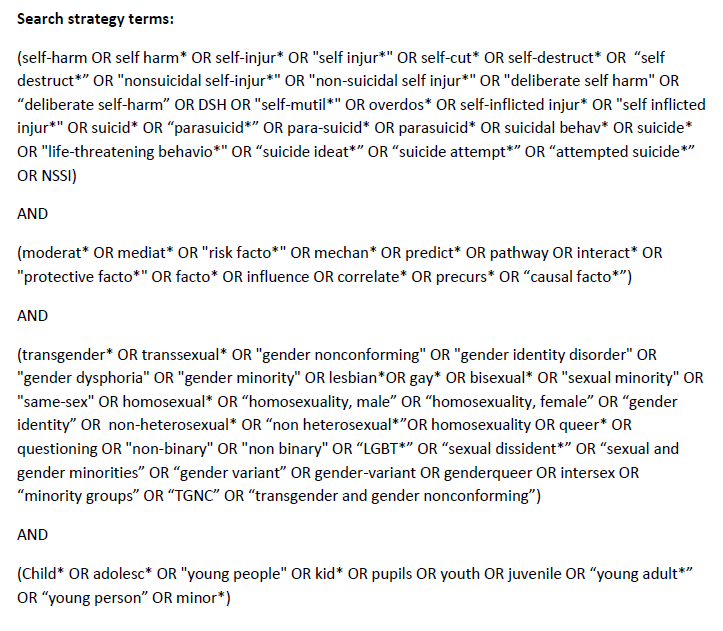

Supplement: S1 Fig — (TIF) [file pone.0245268.s001.tif]

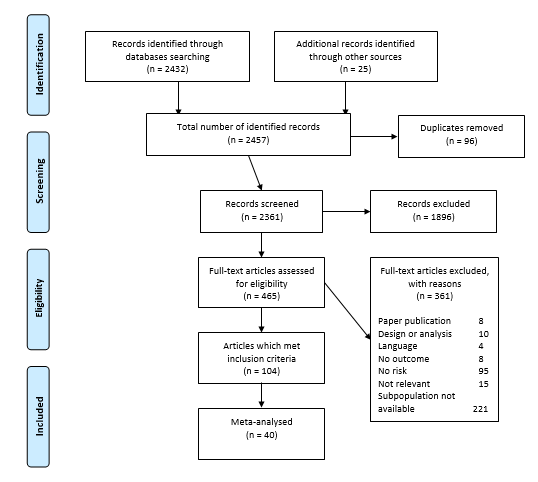

Supplement: S2 Fig — (TIF) [file pone.0245268.s002.tif]

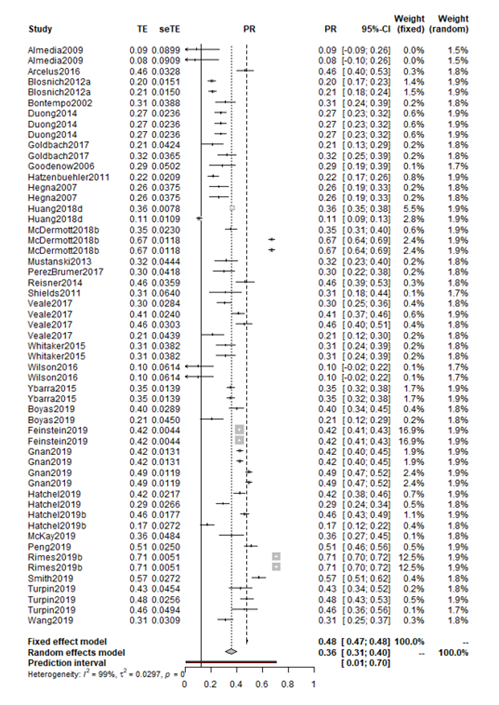

Supplement: S3 Fig — (TIF) [file pone.0245268.s003.tif]

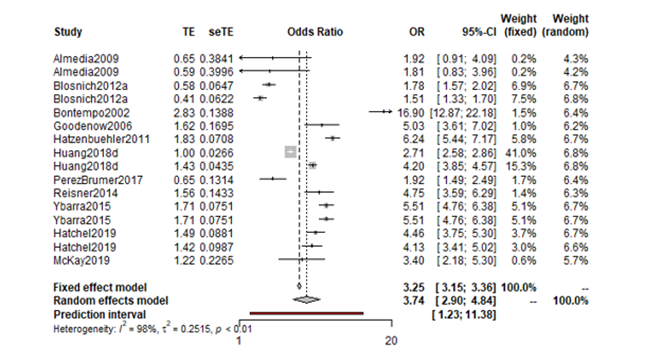

Supplement: S4 Fig — (TIF) [file pone.0245268.s004.tif]

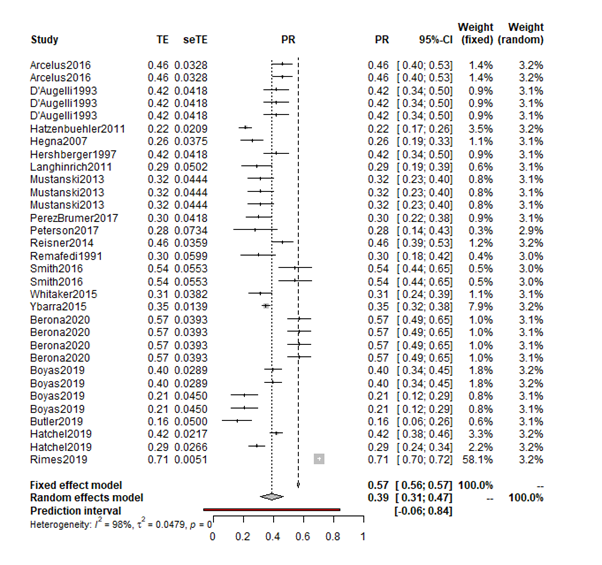

Supplement: S5 Fig — (TIF) [file pone.0245268.s005.tif]

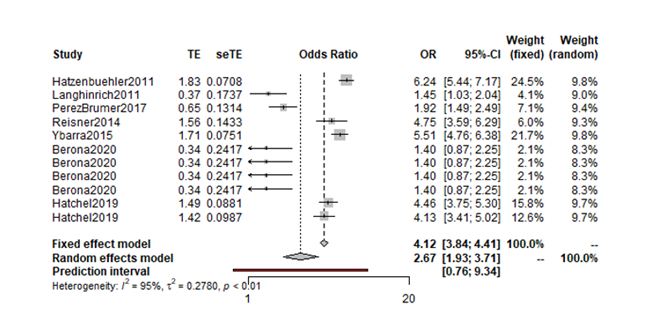

Supplement: S6 Fig — (TIF) [file pone.0245268.s006.tif]
