# Supplementary material for: A systematic review and meta-analysis of victimisation and mental health prevalence among LGBTQ+ young people with experiences of self-harm and suicide
Source: PLoS One. 2021 Jan 22;16(1):e0245268. doi: 10.1371/journal.pone.0245268 (PMC7822285; doi:10.1371/journal.pone.0245268)
Supplement: S1 Table — (DOCX) [file pone.0245268.s007.docx]

**Table 1: Inclusion criteria used during screening process**

| **Inclusion Criteria** | **Exclusion criteria** |
| --- | --- |
| - Peer reviewed studies. - Any geographical location. - English language. - Empirical quantitative studies, following cross-sectional, prospective, longitudinal, cohort and case-control designs. - Participants that have had a measured outcome from the dimension of self-harm and suicide; self-harm (self-harm or injury to self-irrespective of suicidal intent), suicidal ideation (thoughts, plan, death wish), or suicide attempt (individual took an attempt on their life, suicide death). - Studies must consider risks associated with or predictive of self-harm, suicidal ideation, suicidal attempt or death. - Participants must be young people (12-25 years). - Participants that are identified or self-identified as any sexual or gender minority or member of LGBTQ+. | - Non-peer reviewed literature. - Not English language. - Grey literature such as theses, dissertations or conference proceedings. - Articles such as commentaries, reviews, editorial or opinion pieces. - Empirical qualitative studies. - Participants who have no experience of self-harm, suicidal ideation or suicidal attempt. - Sample not aged between 12 and 25 years, e.g. adults 26 years and above or children 12 years and under. - Participants who are identified as heterosexual or not part of sexual or gender minority. |
