# Supplementary material for: A systematic review and meta-analysis of victimisation and mental health prevalence among LGBTQ+ young people with experiences of self-harm and suicide
Source: PLoS One. 2021 Jan 22;16(1):e0245268. doi: 10.1371/journal.pone.0245268 (PMC7822285; doi:10.1371/journal.pone.0245268)
Supplement: S2 Table — (DOCX) [file pone.0245268.s008.docx]

**Table 2: Risks associated with experiences of self-harm or suicide among LGBTQ+ young people: Data unable to be numerically synthesised**

| **Categories of risk** | **LGBQ k=48 N (%)** | **TGNC k=8**  **N (%)** | **LBGTQ+ k=8 N (%)** |
| --- | --- | --- | --- |
| **Demographic variables**  (*e.g. natal gender, age, race*) | 15 (30.6) | 4 (50) | 3 (37.5) |
| **Psychosocial variables**  (*e.g. low self-esteem, dating violence, suicide of friend or family, abuse*) | 31 (63.3) | 4 (50) | 5 (62.5) |
| **Victimisation variables**  (*e.g. LGBTQ hate crime, homophobic bullying, school bullying, cyber bullying*) | 27 (55.1) | 2 (25) | 4 (50) |
| **Mental health difficulties variables**  (*e.g. depression, substance use, bipolar, anxiety*) | 10 (20.4) | 4 (50) | 2 (25) |
| **LGBTQ+ specific variables**  (*e.g. gender-role nonconformity, internalised homophobia, parental rejection, loss of friends due to sexual orientation*) | 13 (26.5) | 2 (25) | 3 (37.5) |
