# Supplementary material for: A systematic review and meta-analysis of victimisation and mental health prevalence among LGBTQ+ young people with experiences of self-harm and suicide
Source: PLoS One. 2021 Jan 22;16(1):e0245268. doi: 10.1371/journal.pone.0245268 (PMC7822285; doi:10.1371/journal.pone.0245268)
Supplement: S3 Table — (DOCX) [file pone.0245268.s009.docx]

**Table 3: Subgroup analyses of victimisation prevalence among LGBTQ+ young people with self-harm or suicidal experiences**

|  | Number of estimates (N) | Prevalence Rate | | 95% CI | Q | I^2^ (%) | ꭓ^2^ | | Q, df , p |
| --- | --- | --- | --- | --- | --- | --- | --- | --- | --- |
| QUALITY RATING | | |  | | | | | Q = 19.50, df = 2, p = 0.01 | |
| Low | 7 | 0.46 | | 0.34-0.58 | 347.88 | 98.3 | 0.02 | |  |
| Moderate | 31 | 0.28 | | 0.24-0.32 | 686.32 | 95.6 | 0.01 | |  |
| High | 17 | 0.45 | | 0.37-0.52 | 4107.33 | 99.6 | 0.02 | |  |
| POPULATION | | |  | | | | | Q = 0.11, df = 1, p = 0.74 | |
| LGBQ | 27 | 0.34 | | 0.27-0.42 | 6282.68 | 99.6 | 0.03 | |  |
| TGNC | 9 | 0.33 | | 0.24-0.41 | 108.99 | 92.7 | 0.01 | |  |
| OUTCOME | | |  | | | | | Q = 12.18, df = 2, p = 0.01 | |
| Self-harm | 10 | 0.39 | | 0.31-0.48 | 353.09 | 97.5 | 0.02 | |  |
| Suicidal ideation | 21 | 0.35 | | 0.33-0.38 | 212.38 | 93.4 | 0.00 | |  |
| Suicidal attempt | 15 | 0.26 | | 0.20-0.31 | 212.38 | 93.4 | 0.01 | |  |
