# Supplementary material for: A systematic review and meta-analysis of victimisation and mental health prevalence among LGBTQ+ young people with experiences of self-harm and suicide
Source: PLoS One. 2021 Jan 22;16(1):e0245268. doi: 10.1371/journal.pone.0245268 (PMC7822285; doi:10.1371/journal.pone.0245268)
Supplement: S4 Table — (DOCX) [file pone.0245268.s010.docx]

**Table 4: Subgroup analyses of mental health difficulties prevalence among LGBTQ+ populations who have experiences of self-harm or suicide**

|  | Number of estimates (N) | Prevalence Rate | 95% CI | Q | I^2^ (%) | ꭓ^2^ | Q, df , p |
| --- | --- | --- | --- | --- | --- | --- | --- |
| QUALITY RATING | | | | | |  | Q = 1.54, df = 2, p = 0.46 |
| Low | 11 | 0.41 | 0.33-0.49 | 122.06 | 91.8 | 0.01 |  |
| Moderate | 17 | 0.36 | 0.31-0.41 | 125.83 | 87.3 | 0.00 |  |
| High | 4 | 0.47 | 0.25-0.69 | 417.38 | 99.3 | 0.05 |  |
| POPULATION | | | | | |  | Q = 2.43, df = 1, p = 0.30 |
| LGBQ | 20 | 0.42 | 0.32-0.53 | 1227.71 | 98.5 | 0.05 |  |
| TGNC | 5 | 0.34 | 0.22-0.45 | 37.56 | 89.4 | 0.01 |  |
| OUTCOME | | | | | |  | Q = 0.41, df = 2, p = 0.82 |
| Self-harm | 3 | 0.38 | 0.20-0.53 | 30.19 | 93.4 | 0.02 |  |
| Suicidal ideation | 8 | 0.40 | 0.35-0.44 | 32.70 | 78.6 | 0.00 |  |
| Suicidal attempt | 19 | 0.38 | 0.31-0.44 | 222.21 | 91.9 | 0.02 |  |
