# Supplementary material for: A systematic review and meta-analysis of victimisation and mental health prevalence among LGBTQ+ young people with experiences of self-harm and suicide
Source: PLoS One. 2021 Jan 22;16(1):e0245268. doi: 10.1371/journal.pone.0245268 (PMC7822285; doi:10.1371/journal.pone.0245268)
Supplement: S2 File — (PDF) [file pone.0245268.s012.pdf]

NOS Case Control

Selection

1) Is the case definition adequate:
    - a) Yes, with independent validation. *
    - b) Yes, e.g. self-reports.
    - c) No description.

2) Representativeness of the cases:
    - a) Consecutive or obviously representative series of cases. *
    - b) Potential for selection biases or not stated.

3) Selection of Controls:
    - a) Community controls. *
    - b) Hospital controls.
    - c) No description.

4) Definition of Controls:
    - a) No history of suicide/self-harm. *
    - b) No description of source.

Comparability

1) Comparability of cases and controls on the basis of the design or analysis:
    - a) Study controls for LGBTQ status. *
    - b) Study controls for LGBTQ and self-harm/suicide. **

Exposure

1) Ascertainment of exposure:
    - a) Secure record (e.g. surgical records). *
    - b) Structured interview where blind to case/control status. *
    - c) Self-report.
    - d) No description.

2) Same method of ascertainment for cases and controls:
    - a) Yes. *
    - b) No.

3) Non-Response rate:
    - a) Same rate for both groups. *
    - b) Non respondents described.
    - c) Rate different and no designation.

NOS Cohort

Selection

1) Representativeness of the exposed cohort:
   - a) Truly representative of the average in target population (all subjects or random sampling). *
   - b) Somewhat representative of the average in the target population (non-random sampling). *
   - c) Selected group of users.
   - d) No description.

2) Selection of the non-exposed cohort:
   - a) Drawn from the same community as the exposed cohort. *
   - b) Drawn from a different source.
   - c) No description.

3) Ascertainment of exposure:
   - a) Secure record (e.g. surgical records). *
   - b) Structured interview. *
   - c) Written self-report.
   - d) No description.

4) Demonstration that outcome of interest was not present at start of study:
   - a) Yes. *
   - b) No.

Comparability

1) Comparability of cohorts on the basis of the design or analysis:
   - a) Study controls for LGBTQ status. *
   - b) Study controls for LGBTQ and self-harm/suicide. **

Outcome

1) Assessment of outcome:
   - a) Independent blind assessment. *
   - b) Record linkage. *
   - c) Self-report.
   - d) No description.

2) Was follow-up long enough for outcomes to occur:
   - a) Yes e.g. 6 months. *
   - b) No.

3) Adequacy of follow up of cohorts:
   - a) Complete follow up - all subjects accounted for. *
   - b) Subjects lost to follow up but rate given (description given). *
   - c) Subjects lost to follow up (no description given).
   - d) No description at all.

NOS Cross Sectional

1) Representativeness of the sample:

a) Truly representative of the average in the target population. (all subjects or random sampling) *

b) Somewhat representative of the average in the target population. (non-random sampling) *

c) Selected group of users.

d) No description of the sampling strategy.

2) Sample size:

a) Justified and satisfactory. *

b) Not justified.

3) Non-respondents:

a) Comparability between respondents and non-respondents characteristics is established, and the response rate is satisfactory. *

b) The response rate is unsatisfactory, or the comparability between respondents and non-respondents is unsatisfactory.

c) No description of the response rate or the characteristics of the responders and the non-responders.

4) Ascertainment of the exposure:

a) Validated measurement tool. **

b) Non-validated measurement tool, but the tool is available or described. *

c) No description of the measurement tool.

Comparability:

1) The subjects in different outcome groups are comparable, based on the study design or analysis. Confounding factors are controlled.

a) study controls for LGBTQ status *

b) study controls for LGBTQ and self-harm/suicide **

Outcome:

1) Assessment of the outcome:

a) Independent blind assessment. **

b) Record linkage. **

c) Self report. *

d) No description.

2) Statistical test:

a) The statistical test used to analyze the data is clearly described and appropriate, and the measurement of the association is presented, including confidence intervals and the probability level (p value). *

b) The statistical test is not appropriate, not described or incomplete.
