# Supplementary material for: A systematic review and meta-analysis of victimisation and mental health prevalence among LGBTQ+ young people with experiences of self-harm and suicide
Source: PLoS One. 2021 Jan 22;16(1):e0245268. doi: 10.1371/journal.pone.0245268 (PMC7822285; doi:10.1371/journal.pone.0245268)
Supplement: S3 File — (DOCX) [file pone.0245268.s013.docx]

**SUPPLEMENTARY RESULTS**

***Supplementary Table A: Characteristics of non-meta-analysed studies***

| **Author (date) country** | **No. total participants** | **No. LGBQ (%)**  **No. TGNC (%)** | **Participant age range (years)** | **Setting** | **Outcome** | **Identified Risks** | **Quality** |
| --- | --- | --- | --- | --- | --- | --- | --- |
| Antonio & Moleiro (2015)^(50)^  Portugal | 211 | LGBQ: 148 (70.1)  TGNC: - | 12-20 | Community | Suicidal ideation | Low social support  Victimisation | Moderate |
| Baams et al., (2015)^(41)^  U.S.A | 876 | LGBQ: 876 (100)  TGNC: - | 15-21 | Community | Suicidal ideation | Coming-out stress  Perceived burdensome  Thwarted belongingness  Victimisation | Low |
| Baiden et al., (2019)^(51)^  U.S.A | 9,693 | LGBQ: 722 (7.5)  TGNC: - | 14-18 | School | Suicidal ideation; Suicidal attempt | Teen dating violence | Moderate |
| Ballard et al., (2017)^(52)^  U.S.A | 1,550 | LGBQ: 155 (10)  TGNC: - | 14-18 | School | Suicidal ideation; Suicidal attempt | Victimisation | Moderate |
| Birkett et al., (2009)^(53)^  U.S.A | 7,376 | LGBQ: 1,118 (15.1)  TGNC: - | 12-14 | School | Suicidal ideation | Negative perception of education  Victimisation | High/Moderate |
| Bostwick et al., (2014)^(18)^  U.S.A | 72,691 | LGBQ: 6,245 (8.6)  TGNC: - | 13-18 | Community | Self-harm;  Suicidal ideation;  Suicidal attempt | Gender (female)  Ethnic minority | Low |
| Burton et al., (2013)^(54)^  U.S.A | 192 | LGBQ: 55 (29)  TGNC: - | 14-19 | Hospital/Clinic | Suicidal ideation; Suicidal attempt | Victimisation | Low |
| Button (2016)^(55)^  U.S.A | 484 | LGBQ: 484 (100)  TGNC: - | 12-18 | School | Suicidal ideation; Suicidal attempt | Age (below 14)  Victimisation | High/Moderate |
| Cenat et al., (2015)^(56)^  Canada | 8,194 | LGBQ: 1,426 (17.4)  TGNC: - | 14-20 | School | Suicidal ideation | Sexuality (bisexual)  Victimisation | Moderate |
| Consolacion et al., (2004)^(16)^  U.S.A | 13,205 | LGBQ: 1,189 (9)  TGNC: - | 12-18 | Community | Suicidal ideation | Gender (female)  Race (white) | Low |
| Coulter et al., (2015)^(57)^  U.S.A | 75,192 | LGBQ: 6,558 (8.7)  TGNC: 175 (0.2) | 18-25 | School | Self-harm; Suicidal ideation | Alcohol-related problems | Moderate |
| Cutuli et al., (2020)^(58)^  U.S.A | 77,559 | LGBQ: 6,903 (8.9)  TGNC: - | 14-18 | School | Suicidal ideation; Suicidal attempt | Homelessness | Moderate |
| D’Augelli et al., (2001)^(59)^  U.S.A | 350 | LGBQ: 350 (100)  TGNC: - | 14-21 | Community | Suicidal attempt | Age (below 16)  Personal homonegativity  Parental rejection  LGB-friends suicide attempts | High/Moderate |
| D’Augelli et al., (2005)^(60)^  U.S.A | 361 | LGBQ: 361 (100)  TGNC: - | 15-19 | Community | Suicidal attempt | Personal homonegativity  Parental discourage of childhood gender atypical behaviour  Parental labelling of sexual orientation  Parental psychological abuse  Family history of suicide  Victimisation | Moderate |
| De Assis et al., (2014)^(61)^  Brazil | 3,205 | LGBQ: 122 (3.8)  TGNC: - | 15-19 | School | Suicidal ideation | Problems with romantic relationship | Low |
| Duncan & Hatzenbuehler (2014)^(62)^  U.S.A | 1,173 | LGBQ: 102 (8.7)  TGNC: - | 14-18 | School | Suicidal ideation; Suicidal attempt | LGBT hate crime – threat  LGBT hate crime – harassment  LGBT hate crime – assault or battery  LGBT hate crime – assault or battery with weapon | Moderate |
| DuRant et al., (1998)^(63)^  U.S.A | 3,886 | LGBQ: 338 (8.7)  TGNC: - | 12-18 | School | Suicidal attempt | Higher number of male sexual partners | Moderate |
| Eisenberg et al., (2016)^(64)^  U.S.A | 122,180 | LGBQ: 6,223 (5.1)  TGNC: - | 13-17 | School | Suicidal attempt | Gender (female)  Low levels of LGBQ peers  Victimisation | Moderate |
| Eisenberg et al., (2019)^(65)^  U.S.A | 2,168 | LGBQ: -  TGNC: 2168 (100) | 14-17 | School | Self-harm;  Suicidal ideation;  Suicidal attempt | Location (rural areas) | Moderate |
| Espelage et al., (2008)^(66)^  U.S.A | 13,921 | LGBQ: 1,997 (1.6)  TGNC: - | 14-18 | School | Suicidal ideation | Victimisation | High/Moderate |
| Espelage et al., (2018)^(67)^  U.S.A | 11,794 | LGBQ: 767 (6.5)  TGNC: 212 (1.8) | 14-18 | School | Suicidal ideation; Suicidal attempt | Teen dating violence  Negative perception of school violence and crime  Victimisation | High/Moderate |
| Fraser et al., (2018)^(68)^  New Zealand | 1,799 | LGBQ: 198 (11)  TGNC: 5 (0.3) | 13-18 | School | Self-harm | Sexuality (bisexual)  Sexuality concerns  Emotional regulation | Moderate |
| Friedman et al., (2006)^(69)^  U.S.A | 96 | LGBQ: 96 (100)  TGNC: - | 18-25 | Community | Suicidal ideation; Suicidal attempt | High femininity during middle school  Masculinity during elementary school  Gender-role nonconformity  Victimisation | Moderate |
| Garofalo et al., (1999)^(70)^  U.S.A | 3,365 | LGBQ: 129 (3.8)  TGNC: - | 14-18 | School | Suicidal attempt | Gender (male)  Drug and alcohol use  Sexual activity risk (against will)  Violence and victimisation risk | Moderate |
| Gibbs & Goldbach (2015)^(71)^  U.S.A | 2,949 | LGBQ: 2944 (99.8)  TGNC: 75 (2.5) | 18-24 | Community | Suicidal ideation | Parental anti-homosexual religious beliefs  Leaving religion of origin due to conflict  Internalised homophobia  Religious upbringing with unresolved conflict | Moderate |
| Grossman & Kerner (1998)^(72)^  U.S.A | 90 | LGBQ: 90 (100)  TGNC: - | 14-21 | Community | Suicidal ideation | Low self-esteem  Emotional distress | Moderate |
| Grossman et al., (2007^)(73)^  U.S.A | 55 | LGBQ: -  TGNC: 55 (100) | 15-21 | Community | Suicidal attempt | Transgender-related suicide negativity  Body esteem – weight  Body esteem – attribution  Other’s evaluation of body and appearance  Parental verbal abuse  Parental physical abuse | Moderate |
| Grossman et al., (2016)^(74)^  U.S.A | 129 | LGBQ: -  TGNC: 129 (100) | 15-21 | Community | Suicidal ideation; Suicidal attempt | Gender (natal female)  Race (white)  High thwarted belongingness  High perceived burdensomeness  Previous experiences of painful and provocative events | Low |
| Halkitis et al., (2018)^(75)^  U.S.A | 665 | LGBQ: 665 (100)  TGNC: - | 18-23 | Community | Suicidal ideation; Suicidal attempt | Loneliness  Low self-esteem | Moderate |
| Hee-Kim et al., (2016)^(76)^  South Korea | 146,621 | LGBQ: 1,270 (0.9)  TGNC: - | 12-17 | School | Suicidal ideation; Suicidal attempt | STDs experience  Violence | Moderate |
| Higgins-Tejera et al., (2019)^(77)^  U.S.A | 10,386 | LGBQ: 926 (8.9)  TGNC: | 16-17 | School | Suicidal ideation | Victimisation | Moderate |
| Hightow-Weidman et al., (2011)^(78)^  U.S.A | 351 | LGBQ: 351 (100)  TGNC: 16 (4.6) | 13-24 | Community | Suicidal attempt | Sexual minority and disability status | Moderate |
| Huang et al., (2018a)^(49)^  China | 123,459 | LGBQ: 6685 (5)  TGNC: - | 12-18 | School | Suicidal attempt | Poor sleep quality | Moderate |
| Huang et al., (2018b) ^(79)^  China | 72,409 | LGBQ: 15,066 (19.9)  TGNC: - | 12-20 | School | Suicidal ideation;  Suicidal attempt | Obesity | Moderate |
| King et al., (2018)^(80)^  U.S.A | 11,364 | LGBQ: 730 (6.4)  TGNC: - | 14-18 | School | Suicidal ideation | Low school connectedness  Victimisation | Moderate |
| Lardier et al., (2017)^(81)^  U.S.A | 538 | LGBQ: 70 (13)  TGNC: - | 14-18 | Community | Suicidal ideation | Depressive symptoms  Victimisation | Moderate |
| LeVasseur et al., (2013)^(82)^  U.S.A | 11,887 | LGBQ: 939 (7.9)  TGNC: - | 14-18 | School | Suicidal attempt | Victimisation | Moderate |
| Li et al., (2019)^(83)^  China | 1,810 | LGBQ: 310 (17.1) | 15-18 | School | Self-harm | Adverse childhood experiences | Moderate |
| Liu & Mustanski (2012)^(84)^  U.S.A | 246 | LGBQ: 244 (99.2)  TGNC: 20 (8.1) | 16-20 | Community | Self-harm; Suicidal ideation | Gender (female)  Gender nonconformity  History of attempted suicide  Depressive symptoms  Impulsivity  Low social support  Sensation-seeking  Hopelessness  Victimisation | High |
| Lytle et al., (2018)^(85)^  U.S.A | 203 | LGBQ: 73 (36)  TGNC: 18 (8.9) | 18-24 | Community | Suicidal ideation; Suicidal attempt | Sex assigned at birth  Depression/anxiety  Friend suicide attempt or complete  Family suicide attempt or complete  Less perceived family support | High/Moderate |
| Marx et al., (2019)^(86)^  U.S.A | 16,292 | LGBQ: 2,786 (17.1)  TGNC: 610 (3.7) | 14-18 | School | Suicidal ideation | Problematic drug use  Victimisation | Moderate |
| Mendoza-Pérez et al., (2019)^(43)^  Mexico | 23,496 | LGBQ: 2,350 (10)  TGNC: - | 14-19 | School | Suicidal ideation; Suicidal attempt | Negative attitudes towards homosexuality  Violent experience | Moderate |
| Mustanski et al., (2010)^(87)^  U.S.A | 246 | LGBQ: 241 (98)  TGNC: 20 (8.1) | 16-20 | Community | Suicidal attempt | Gender (female) | Moderate |
| Mustanski et al., (2014)^(88)^  U.S.A | 16,977 | LGBQ: 1,185 (7)  TGNC: - | 13-18 | Community | Suicidal attempt | Cocaine use  Feelings of sadness  Intimate partner violence  Victimisation | Moderate |
| Palm et al., (2016)^(89)^  Sweden | 1,051 | LGBQ: 105 (10)  TGNC: - | 15-22 | Community | Suicidal ideation; Suicidal attempt | Multiple victimisations | Moderate |
| Poteat et al., (2009)^(90)^  Unclear | 14,439 | LGBQ: 3,321 (23)  TGNC: - | 14-19 | Community | Suicidal ideation | Victimisation | Moderate |
| Proctor & Groze (1994)^(91)^  Multiple countries | 221 | LGBQ: 221 (100)  TGNC: - | - | Community | Suicidal attempt | Depressive symptoms  Poor parental relations  Poor school performance  Low self-esteem | Moderate |
| Puckett et al., (2017)^(92)^  U.S.A | 61 | LGBQ: 61 (100)  TGNC: - | 14-23 | Community | Suicidal attempt | Gender (female)  Loss of friends due to sexual orientation  Guilt/shame due to sexual orientation  Internalised homophobia  Psychological maltreatment from caregivers | Moderate |
| Remafedi (2002)^(93)^  U.S.A | 255 | LGBQ: 255 (100)  TGNC: - | 15-25 | Community | Suicidal attempt | Race (black/African-American)  Location (urban areas)  Fewer years in education  Lower enrolment in school | Moderate |
| Rimes et al., (2017)^(94)^  U.K | 677 | LGBQ: 622 (91.9)  TGNC: 677 (100) | 16-25 | Community | Self-harm | Gender (natal female) | Moderate |
| Rotheram-Borus et al., (1994)^(95)^  U.S.A | 131 | LGBQ: 127 (96.9)  TGNC: - | 14-19 | Community | Suicidal attempt | Dropped out of school  Lived outside of family home  Came out to parents  Came out to siblings  Parental discovery of sexuality  Friend or family suicide attempt | High/Moderate |
| Russell & Joyner (2001)^(96)^  U.S.A | 11,940 | LGBQ: 836 (7)  TGNC: - | 12-15 | School | Suicidal ideation; Suicidal attempt | Alcohol abuse  Depression  Friend or family suicide attempt  Hopelessness  Victimisation | Moderate |
| Ryan et al., (2009)^(97)^  U.S.A | 224 | LGBQ: 224 (100)  TGNC: - | 21-25 | Community | Suicidal attempt | Family rejection | Moderate |
| Savin-Williams & Ream (2003)^(98)^  Multiple countries | 732 | LGBQ: 727 (99.3)  TGNC: - | 13-25 | Community | Suicidal attempt | Younger age of first sexual experience with male  More male partners  Mastery orientation  Earlier age at first disclosure  Higher rate of disclosure  Less acceptance of their sexual orientation  Alcohol use  Hard drug use  Depressive symptoms  Low self-esteem  Greater willingness to engage in risky sex  Victimisation | Moderate |
| Scheer et al., (2019)^(99)^  U.S.A | 7,532 | LGBQ: 1089 (14.5)  TGNC: - | 14-18 | School | Suicidal ideation; Suicidal attempt | Sexual violence | Moderate |
| Shearer et al., (2018)^(100)^  U.S.A | 129 | LGBQ: 41 (31.9)  TGNC: - | 12-18 | Community | Suicidal attempt | Religiosity  More religious parents | High/Moderate |
| Taliaferro et al., (2018a)^(101)^  U.S.A | 922 | LGBQ: 922 (100)  TGNC: - | 14-18 | School | Suicidal attempt | Same-sex sexual experience  Binge drinking  Marijuana use  Substance use  Relationship violence  Multiple sexual partners  Victimisation | Moderate |
| Taliaferro et al., (2019)^(102)^  U.S.A | 1,635 | LGBQ: -  TGNC: 1,635 (100) | 14-17 | School | Self-harm; Suicidal attempt | Gender (natal female)  Mental health problem  Positive screen for depression  Alcohol use  Marijuana use  Physical or sexual abuse  Relationship violence  Run away from home  Bullying perpetrator  Victimisation | Moderate |
| Teasdale & Bradley-Engen (2010)^(103)^  U.S.A | 11,911 | LGBQ: 787 (7)  TGNC: - | 12-18 | School | Suicidal ideation; Suicidal attempt | Gender (female)  Location (suburban)  Suicide of close friend  Suicide of family member  Run away from home  Victimisation | Moderate |
| Thoma & Huebner (2013)^(104)^  U.S.A | 276 | LGBQ: 276 (100)  TGNC: 22 (8) | 14-19 | Community | Suicidal ideation | Antigay discrimination  Greater levels of perceived discrimination  Racial discrimination | Moderate |
| Waldo et al., (1998)^(105)^  U.S.A | 248 | LGBQ: 248 (100)  TGNC: - | 15-21 | Community | Suicidal ideation; Suicidal attempt | Low self-esteem  Psychological distress  Victimisation | Moderate/Low |
| Walls et al., (2008)^(106)^  U.S.A | 142 | LGBQ: 142 (100)  TGNC: - | 14-21 | Community | Suicidal ideation; Suicidal attempt | Gender (female)  Methamphetamine use  Hopelessness  Homelessness  Victimisation | High/Moderate |
| Walls et al., (2010)^(107)^  U.S.A | 265 | LGBQ: 265 (100)  TGNC: 13 (4.9) | 13-22 | Community | Self-harm | Younger age  Sexuality (lesbian)  Sexuality (bisexual)  Gender identity (transgender)  Level of outness  Depression  History of attempted suicide  Daily smoking  Inhalant use  Homelessness  Friend suicide attempt or complete | High/Moderate |
| Yadegarfard et al., (2013)^(108)^  Thailand | 190 | LGBQ: -  TGNC: 190 (100) | 15-25 | Community | Suicidal ideation | Number of sexual partners | Moderate |
| Yadegarfard et al., (2014)^(109)^  Thailand | 260 | LGBQ: -  TGNC: 129 (49.6) | 15-25 | Community | Suicidal ideation; Suicidal attempt | Family rejection  Depression  PANSI-negative  Low social support  Loneliness | Moderate |

**Supplementary table B: Characteristics of meta-analysed studies**

| **Author (date) country** | **No. total participants** | **No. LGBQ (%)**  **No. TGNC (%)** | **Participant age range (years)** | **Setting** | **Outcome** | **Quality** | **Multiple Reports** |
| --- | --- | --- | --- | --- | --- | --- | --- |
| Almedia el al., (2009)^(110)^  U.S.A | 1,032 | LGBQ: 93 (9)  TGNC: 17 (1.7) | 13-19 | School | Self-harm;  Suicidal ideation | High/Moderate |  |
| Arcelus et al., (2016)^(111)^  U.K. | 268 | LGBQ: -  TGNC: 268 (100) | - | Hospital/Clinic | Self-harm | Moderate |  |
| Berona et al., (2020)^(112)^  U.S.A | 285 | LGBQ: 119 (41.8)  TGNC: 7 (2.5) | 13-25 | Hospital/Clinic | Self-harm;  Suicidal ideation;  Suicidal attempt | Low/Moderate |  |
| Blosnich et al., (2012)^(113)^  U.S.A | 11,046 | LGBQ: 773 (7)  TGNC: - | 18-24 | Community | Self-harm;  Suicidal ideation;  Suicidal attempt | High/Moderate |  |
| Bontempo et al., (2002)^(114)^  U.S.A | 9,188 | LGBQ: 315 (3.4)  TGNC: - | 14-18 | School | Suicidal attempt | Moderate |  |
| Boyas et al., (2019)^(16)^  U.S.A | 451 | LGBQ: 451 (100)  TGNC: - | 12-18 | School | Suicidal ideation; Suicidal attempt | Low/Moderate |  |
| Butler et al., (2019)^(115)^  U.K. | 8,440 | LGBQ: -  TGNC: 282 (3.3) | 13-17 | School | Self-harm | Moderate |  |
| D’Augelli et al.,(1993)^(42)^  U.S.A | 194 | LGBQ: 194 (100)  TGNC: - | 15-21 | Community | Suicidal attempt | Moderate | Hershberger et al., (1997)^(46)^ |
| Duong & Bradshaw (2014)^(116)^  U.S.A | 951 | LGBQ: 951 (100)  TGNC: - | 14-18 | School | Suicidal attempt | Moderate |  |
| Feinstein et al., (2019)^(117)^  U.S.A | 18,515 | LGBQ: 18,515 (100)  TGNC: - | 14-18 | School | Suicidal attempt | High/Moderate |  |
| Gnan et al., (2019)^(118)^  U.K. | 1,948 | LGBQ: 1927 (98.9)  TGNC: 214 (10.9) | 16-25 | Community | Self-harm;  Suicidal ideation;  Suicidal attempt | High/Moderate |  |
| Goldbach et al., (2017)^(119)^  U.S.A | 346 | LGBQ: 346 (100)  TGNC: - | 14-17 | Community | Self-harm; Suicidal ideation | Moderate |  |
| Goodenow et al., (2006)^(120)^  U.S.A | 3,637 | LGBQ: 202 (5.6)  TGNC: - | 14-18 | School | Suicidal attempt | Low |  |
| Hatchel et al., (2019)^(121)^  U.S.A | 4,867 | LGBQ: 713 (14.6)  TGNC: 129 (1.5) | 12-18 | School | Suicidal ideation; Suicidal attempt | High/Moderate |  |
| Hatchel et al., (2019b)^(122)^  U.S.A | 934 | LGBQ: 769 (82.3)  TGNC: 60 (6.4) | 14-18 | School | Suicidal ideation; Suicidal attempt | High/Moderate |  |
| Hatzenbuehler (2011)^(123)^  U.S.A | 31,852 | LGBQ: 1,413 (4.4)  TGNC: - | 16-17 | School | Suicidal attempt | Moderate |  |
| Hegna &Wichstrøm (2007)^(124)^  Norway | 407 | LGBQ: 407 (100)  TGNC: - | 16-25 | Community | Suicidal attempt | Moderate |  |
| Huang et al., (2018a)^(47)^  China | 123,459 | LGBQ: 6685 (5)  TGNC: - | 12-18 | School | Suicidal attempt | Moderate | Huang et al., 2018d^(49)^ |
| Langhinrichsen-Rohling et al., (2011)^(125)^  U.S.A | 1,533 | LGBQ: 200 (13)  TGNC: - | 13-18 | Community | Suicidal ideation; Suicidal attempt | Moderate |  |
| McDermott et al., (2018)^(126)^ U.K. | 789 | LGBQ: 789 (100)  TGNC: 178 (22.6) | 13-25 | Community | Self-harm; Suicidal ideation; Suicidal attempt | Low |  |
| McKay et al., (2019)^(127)^  U.S.A | 485 | LGBTQ: 175 (36.1) | 14-21 | Community | Suicidal ideation; Suicidal attempt | Moderate |  |
| Mustanski et al., (2013)^(128)^  U.S.A | 237 | LGBQ: 237 (100)  TGNC: 21 (8.9) | 16-20 | Community | Suicidal attempt | Low |  |
| Peng et al., (2019)^(129)^  China | 385 | LGBQ: -  TGNC: 385 (100) | 12-18 | Community | Suicidal ideation | High/Moderate |  |
| Perez-Brumer et al., (2017)^(130)^  U.S.A | 25,493 | LGBQ: 2,440 (9.6)  TGNC: 280 (1.1) | 14-18 | School | Suicidal ideation | High/Moderate |  |
| Peterson et al., (2017)^(131)^  U.S.A | 96 | LGBQ: 96 (100)  TGNC: 96 (100) | 12-22 | Hospital/Clinic | Suicidal attempt | Moderate |  |
| Reisner et al., (2014)^(132)^  U.S.A | 3,131 | LGBQ: 225 (7.2)  TGNC: - | 14-18 | School | Self-harm; Suicidal attempt | High |  |
| Remafedi et al., (1991)^(23)^  U.S.A | 137 | LGBQ: 137 (100)  TGNC: - | 14-21 | Community | Suicidal attempt | Moderate |  |
| Rimes et al., (2019)^(133)^  U.K. | 3,275 | LGBQ: 3275 (100)  TGNC: - | 16-25 | Community | Suicidal ideation; Suicidal attempt | High/Moderate |  |
| Shields et al., (2011)^(134)^  U.S.A | 2,154 | LGBQ: 2,154 (100)  TGNC: - | 14-18 | School | Suicidal ideation | Moderate |  |
| Smith et al., (2016)^(135)^  U.S.A | 68 | LGBQ: 68 (100)  TGNC: - | 16-24 | Community | Suicidal ideation | Moderate |  |
| Smith et al., (2019)^(136)^  U.S.A | 252 | LGBQ: 179 (71)  TGNC: 73 (29) | 14-15 | Community | Self-harm; Suicidal ideation; Suicidal attempt | Low/Moderate |  |
| Taliaferro et al., (2016)^(137)^  U.S.A | 77,758 | LGBQ: 4,960 (6.5)  TGNC: - | 14-18 | School | Self-harm; Suicidal ideation; Suicidal attempt | Moderate |  |
| Taliaferro et al., (2018b)^(138)^  U.S.A | 2,168 | LGBQ: -  TGNC: 2,168 (100) | 14-17 | School | Self-harm | Moderate |  |
| Turpin et al., (2019)^(139)^  U.S.A | 924 | LGBQ: 691 (74.8)  TGNC: - | 14-18 | School | Suicidal ideation | High/Moderate |  |
| Veale et al., (2017)^(140)^  Canada | 923 | LGBQ: -  TGNC: 923 (100) | 14-25 | Community | Self-harm; Suicidal attempt | Moderate |  |
| Wang et al., (2019)^(141)^  Taiwan | 500 | LGBQ: 500 (100)  TGNC: - | 20-25 | Community | Suicidal ideation; Suicidal attempt | High |  |
| Whitaker et al., (2015)^(142)^  U.S.A | 356 | LGBQ: 356 (100)  TGNC: 64 (16.9) | 14-18 | School | Suicidal ideation | High/Moderate |  |
| Wilson et al., (2016)^(143)^  U.S.A | 216 | LGBQ: 147 (68.1)  TGNC: 105 (48.6) | 16-24 | Community | Suicidal ideation | Moderate |  |
| Ybarra et al., (2015)^(144)^  U.S.A | 5,542 | LGBQ: 2,162 (39)  TGNC: 442 (7.8) | 13-18 | Community | Suicidal ideation | Moderate |  |

*
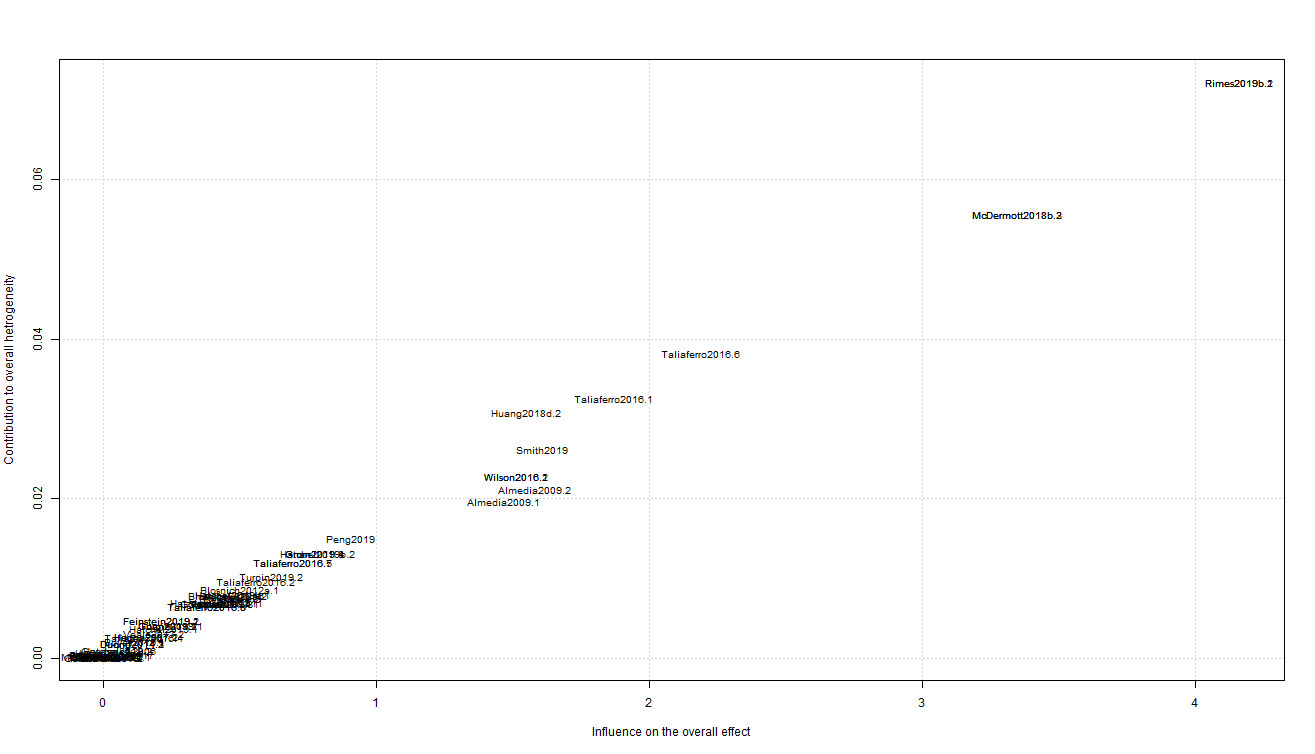
***SUPPLEMENTARY FIGURES**

***Supplementary Figure A: Baujat chart of overall victimisation prevalence***


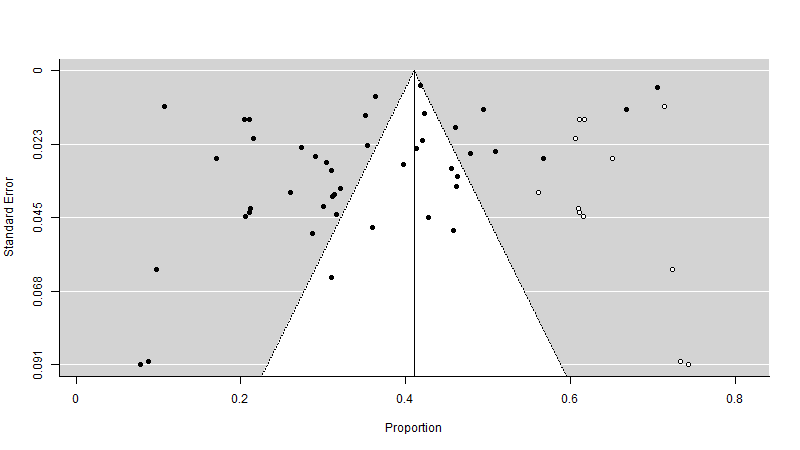


***Supplementary Figure B: Victimisation prevalence funnel plot***


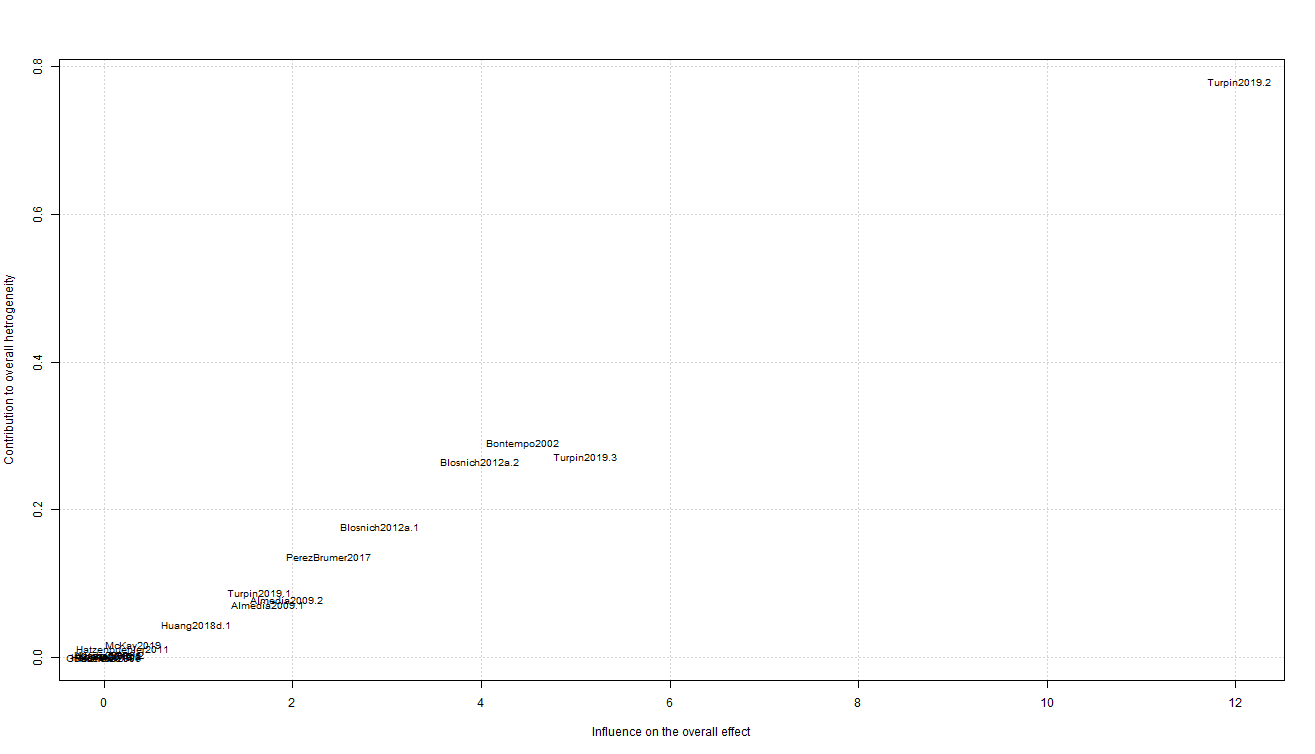


***Supplementary Figure C: Baujat chart of victimisation odds ratio***


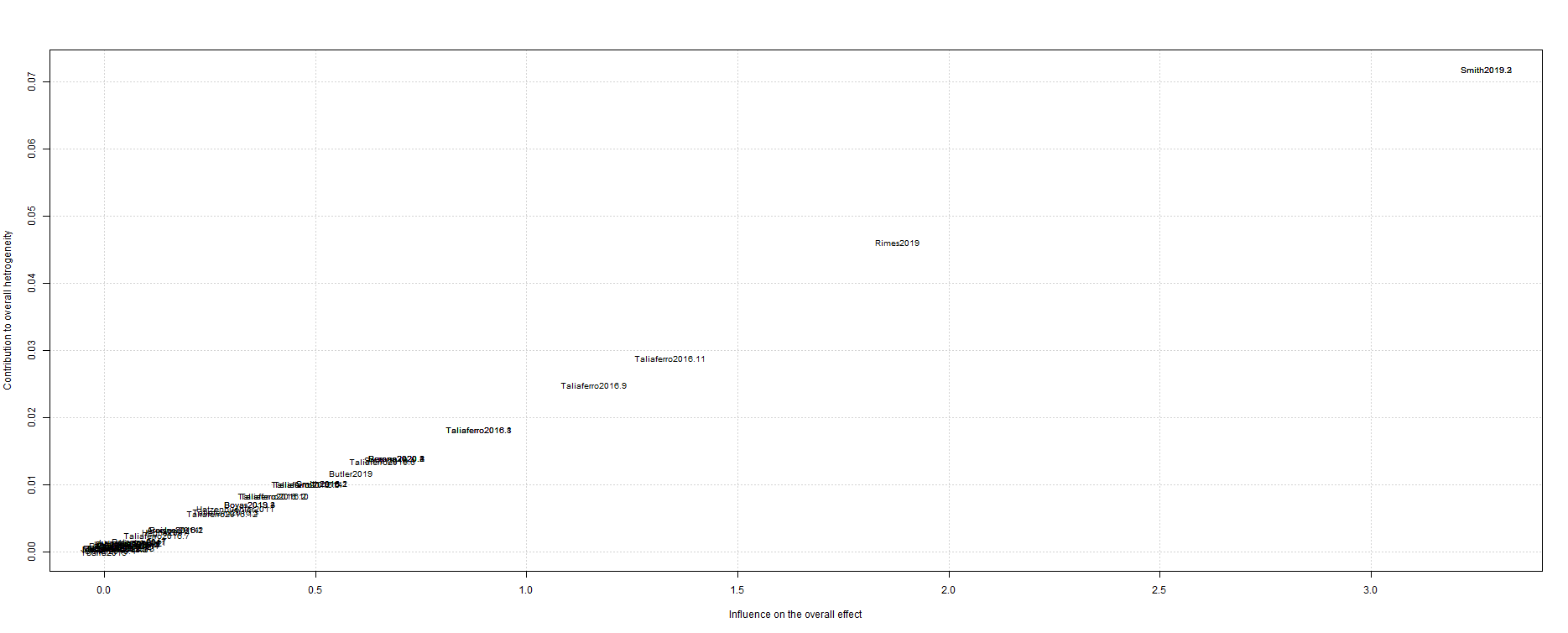


***Supplementary Figure D: Baujat chart of mental health difficulties prevalence***


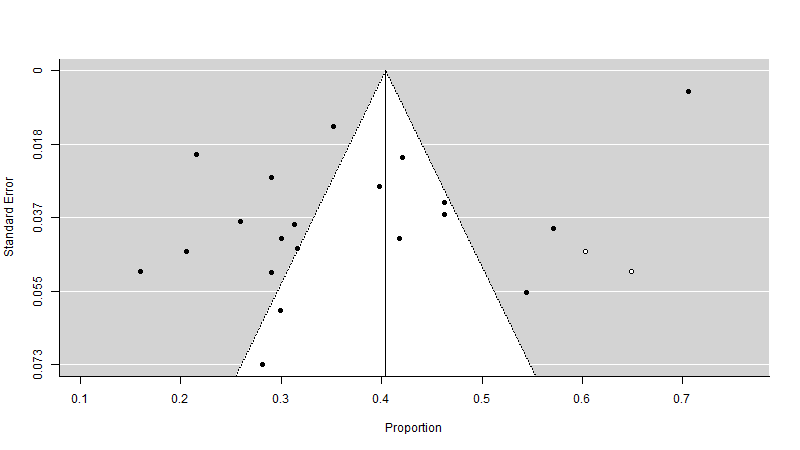


***Supplementary Figure E: Mental health difficulties prevalence funnel plot***
