# Supplementary material for: A systematic review and meta-analysis of victimisation and mental health prevalence among LGBTQ+ young people with experiences of self-harm and suicide
Source: PLoS One. 2021 Jan 22;16(1):e0245268. doi: 10.1371/journal.pone.0245268 (PMC7822285; doi:10.1371/journal.pone.0245268)
Supplement: Attachment — Submitted filename: Response to reviewers.docx [file pone.0245268.s014.docx]

**Response to reviewers:**

1. Please ensure that your manuscript meets PLOS ONE's style requirements, including those for file naming. The PLOS ONE style templates can be found at

https://journals.plos.org/plosone/s/file?id=wjVg/PLOSOne_formatting_sample_main_body.pdf and

https://journals.plos.org/plosone/s/file?id=ba62/PLOSOne_formatting_sample_title_authors_affiliations.pdf

**Thank you for highlighting these changes, this has now been completed.**

2. Please include the full electronic search strategy in the Methods section of the manuscript.

**This has now been added see figure 1, search strategy.**

3. Thank you for stating the following at the end of your manuscript:

'FUNDING STATEMENT
This work was supported by the Economic and Social Research Council for funding this research. The funder of the study had no role in study design, data collection, data analysis, data interpretation, or writing of the report. The corresponding author had full access to all the data in the study and had final responsibility for the decision to submit for publication.'

We note that you have provided funding information that is not currently declared in your Funding Statement. However, funding information should not appear in the Acknowledgments section or other areas of your manuscript. We will only publish funding information present in the Funding Statement section of the online submission form.

Please remove any funding-related text from the manuscript and let us know how you would like to update your Funding Statement. Currently, your Funding Statement reads as follows:

'No - The funders had no role in study design, data collection and analysis, decision to publish, or preparation of the manuscript.'

**This statement has now been removed from the manuscript.**

**We would be happy to have the following funding statement;**

**“This project was funded as part of an Economic and Social Research Council grant on the Doctoral Training Pathway. The lead author, A. Jess Williams, receives a student stipend from the ESRC. The funders had no role in study design, data collection and analysis, decision to publish, or preparation of the manuscript.”**

b. Please include your amended statements within your cover letter; we will change the online submission form on your behalf.

**I appreciate this, we are happy to keep the Funding Statement as stands**.

4. Please include captions for your Supporting Information files at the end of your manuscript, and update any in-text citations to match accordingly. Please see our Supporting Information guidelines for more information: [http://journals.plos.org/plosone/s/supporting-information](about:blank)

**This has now been completed, thank you for highlighting this.**

**Reviewer comment**

Please use the space provided to explain your answers to the questions above. You may also include additional comments for the author, including concerns about dual publication, research ethics, or publication ethics. (Please upload your review as an attachment if it exceeds 20,000 characters)

Reviewer #1: This is an interesting meta-analysis on socially and clinically relevant issues on the border of psychiatry and psychology. In my opinion, the presented analysis was carried out correctly and the methodology has been described sufficiently. The authors performed a careful review according to the PRISMA guidelines and the registered and previously published study protocol. The study was carefully thought out, thanks to which the set goals were achieved. The description of the results is correct, and the discussion interesting and, like the other parts of this article, they do not require major corrections.
I would only suggest to include some information (in the introduction) on the global suicide rate among young people (generally, regardless of orientation) and their causes (e.g. according to WHO reports). In addition, please add keywords that were used in the database search.

**Thank you for your kind review. We really appreciate the time and effort which has been taken to review this manuscript.**

- **Per your suggestion we have added information regarding adolescent suicide rates; lines 51-52.**
- **Risks associated with young people generally was previously included, however we have made this clearer following your comments; lines 64-69.**
- **Search terms and derivatives have been included within the search strategy section; lines 112-114 and we have highlighted that for full search term details readers can view figure 1.**
